# Mapping of chromatin architecture and enhancer-promoter interactions in the cochlea

**DOI:** 10.3389/fmolb.2025.1683964

**Published:** 2025-10-15

**Authors:** Tuba Ege, Celia R. Bloom, Mi Zhou, Huizhan Liu, Litao Tao

**Affiliations:** Biomedical Sciences Department, School of Medicine, Creighton University, Omaha, NE, United States

**Keywords:** 3D genome organization, micro-C, chromatin architecture, enhancer-promoterinteractions, non-coding mutation, hearing loss

## Abstract

**Introduction:**

Chromatin interactions, particularly those between promoters and distal enhancers, enable precise gene regulation in specialized tissues, like the cochlea in the inner ear. Disruptions in these long-range interactions between enhancers and gene promoters are linked to hereditary hearing loss. For many genes important to cochlear development and function, the distal regulatory elements that control their expression remain unknown. Identifying these elements and studying their regulatory roles is challenging due to their distance from target genes and the spatial complexity of chromatin architecture.

**Methods:**

To address this, we employed Micro-C, a high-resolution chromatin conformation capture technique for mapping chromatin interactions, to construct a cochlea-specific chromatin interaction map. We then integrated epigenomic and transcriptomic data to interpret enhancer-promoter interactions involved in gene regulation.

**Results:**

Our analysis revealed unbiased tissue-specific long-range interactions, and some of those interactions overlapped with disease-associated deletions and active regulatory elements, such as the *NR2F1* locus, which is involved in Bosch-Boonstra-Schaaf optic atrophy syndrome (BBSOAS), and the *DLX5/6* locus, which is linked to Split-Hand/Foot Malformation Type 1 (SHFM1), suggesting that structural variants disrupting local chromatin architecture cause transcriptional dysregulation.

**Discussion:**

This study establishes a high-resolution interaction map of the cochlea, demonstrating how non-coding variants can impair tissue-specific gene regulation in hearing loss. Our dataset provides a foundational resource for analyzing hereditary hearing loss mutations and investigating transcriptional regulation in the cochlea.

## 1 Introduction

The complex cellular composition and specialized sensory functions of the inner ear, including both auditory and vestibular organs, require tight regulation and precision of gene expression in a spatiotemporal manner. The higher-order chromatin structures, essential for precise transcriptional regulation of genes for development and functions, remain poorly understood in inner ear organs, including the cochlea (Shi et al., 2024). Eukaryotic genomes are hierarchically compacted into chromatin, from nucleosomes to chromosome territories, through complex genetic and epigenetic mechanisms (Bolzer et al., 2005; Gibcus and Dekker, 2013). This spatial organization of chromatin influences gene regulation, genome stability, and cell identity (Dekker and Misteli, 2015; Dixon et al., 2015). Long-range chromatin interactions, mediated by multicomponent complexes, facilitate communication between distant genomic elements, including enhancer-promoter interactions that are essential for gene transcription (Miele and Dekker, 2008; Lieberman-Aiden et al., 2009; Visel et al., 2009; Deng and Blobel, 2010; Dean, 2011; Rao et al., 2014). These interactions are often stabilized within topologically associating domains (TADs), bringing enhancers into close proximity with their target promoters for efficient and precise gene control (Mora et al., 2015; Chen et al., 2021). Enhancers are a class of cis-regulatory elements (CREs) that function by binding specific transcription factors (TFs) to facilitate gene expression. These elements are traditionally difficult to study due to their methodologically elusive nature. Enhancers can regulate genes from distances spanning hundreds of kilobases to megabases (Preissl et al., 2023). This regulatory relationship is further complicated by the fact that multiple enhancers may regulate a single gene, or a single enhancer may control the expression of multiple genes. In some cases, enhancers form spatial clusters known as multi-enhancer hubs and work together to coordinate gene expression, sometimes even involving enhancers from different chromosomes (Uyehara and Apostolou, 2023). Disruptions to these chromatin interactions can misregulate gene expression, contributing to developmental defects, morphological abnormalities, and sensory disorders, including hearing loss (Bademci et al., 2020). Genome-wide studies have predicted numerous putative enhancers in vertebrates; however, only a small fraction have been directly linked to their target genes or functional phenotypic consequences. Filling this knowledge gap is particularly critical for understanding hereditary hearing loss, where approximately half of all cases present no mutations in gene coding sequences or proximal promoter regions and are therefore likely to be occurring in relatively poorly annotated non-coding regions of the genome. Further complicating this challenge, the paucity of inner ear cells and the difficulty in accessing inner ear organs in their bony labyrinth structure hinder the identification of disease-causing regulatory variants and the functional validation of candidate enhancers (Wong et al., 2020; Tao et al., 2021).

To analyze complex chromatin interactions, chromosome conformation capture-based methods (3C-based), including 3C, 4C, 5C, and Hi-C, have been developed to profile spatial genome organization. While 3C, 4C, and 5C are targeted and require prior knowledge of specific loci, Hi-C and its high-resolution adaptation, Micro-C, enable the unbiased mapping of genome-wide interactions without prior knowledge of interacting elements (Dekker and Mirny, 2013; Sun et al., 2024). The improved resolution and sensitivity of these methods help define TADs and chromatin looping (Hsieh et al., 2020). While Hi-C and related techniques have advanced our understanding of genome structure, their limited resolution, uneven fragment sizes, and high background noise make it difficult to detect fine-scale regulatory interactions in specialized tissues like the cochlea. Micro-C overcomes these issues by using micrococcal nuclease (MNase) to fragment chromatin into individual nucleosomes in a motif-independent manner. This approach generates consistent fragment sizes (100–200 bp), improves genome-wide coverage, and enables analysis at finer resolution than conventional Hi-C, which is critical for identifying cochlea-specific enhancer-promoter interactions (Hsieh et al., 2020; Akgol Oksuz et al., 2021; Lee et al., 2022). In this study, we focused on 5–10 kb contact maps, which provided an appropriate balance between resolution and signal-to-noise. This resolution also enables a closer examination of long-range chromatin interactions, facilitating the scanning of nearby regions by promoters and enhancers to identify their interaction partners.

Here, we apply Micro-C to the postnatal mouse cochlea to generate a high-resolution chromatin interaction map, identifying enhancer-promoter loops at disease-relevant loci in the cochlea. Integrating Micro-C with cochlear epigenomic and transcriptomic data (ATAC-seq, H3K27ac, H3K4me1, CTCF), we demonstrate that disease-associated structural variants overlap key loop anchors, suggesting a mechanism by which mutations in distal non-coding regions could disrupt chromatin architecture and gene regulation in hearing disorders.

To demonstrate the power of Micro-C to identify fine-scale chromatin architecture in the cochlea, we focused on two neurodevelopmental disorders, Bosch-Boonstra-Schaaf optic atrophy syndrome (BBSOAS) and Split-Hand/Foot Malformation Type 1 (SHFM1), where long-range enhancer dysregulation is implicated in auditory dysfunction. BBSOAS, caused by haploinsufficiency of the *NR2F1* gene, which is critical for auditory system development, is characterized by optic atrophy and often includes sensorineural hearing loss (Brown et al., 2009; Chen et al., 2016). While the human *NR2F1* regulatory landscape is poorly defined, mouse studies indicate a critical distal enhancer within the *Mctp1* gene regulating *Nr2f1* expression in the cochlea. Deletion of this enhancer, leaving the coding sequence intact, reduces *Nr2f1* expression by 50% and causes hearing loss, demonstrating the pathogenic potential of enhancer disruption (Tang et al., 2006; Tarchini et al., 2018). SHFM1, a congenital limb malformation often accompanied by sensorineural hearing loss, frequently lacks *DLX5* or *DLX6* coding region mutations (Birnbaum et al., 2012a; Allen et al., 2014). Instead, structural rearrangements disrupt distal enhancers found within the *DYNC1I1* gene that are essential for proper *DLX5/6* expression (Brown et al., 2010). In humans, deletions, duplications, or inversions that disrupt the physical proximity between *DYNC1I1* enhancers (exons 15–17) and the *DLX5/6* locus are associated with limb and inner ear abnormalities (Birnbaum et al., 2012b; Ambrosetti et al., 2023). Mouse models confirm these exonic regions function as enhancers; their deletion downregulates *Dlx5/6*, causing inner ear anomalies that include cochlear malformations and hearing loss (Birnbaum et al., 2012b). However, the direct evidence of the tissue-specific chromatin loops connecting these critical enhancers to their target promoters within the cochlea has yet to be established. This information gap prevents our understanding of how their disruption causes tissue-specific transcriptional dysregulation and hearing loss. Our work elucidates the 3D regulatory landscape of the auditory system and establishes a framework for understanding the functional impact of non-coding mutations.

## 2 Results

### 2.1 Sample preparation and data processing

**FIGURE 1:**
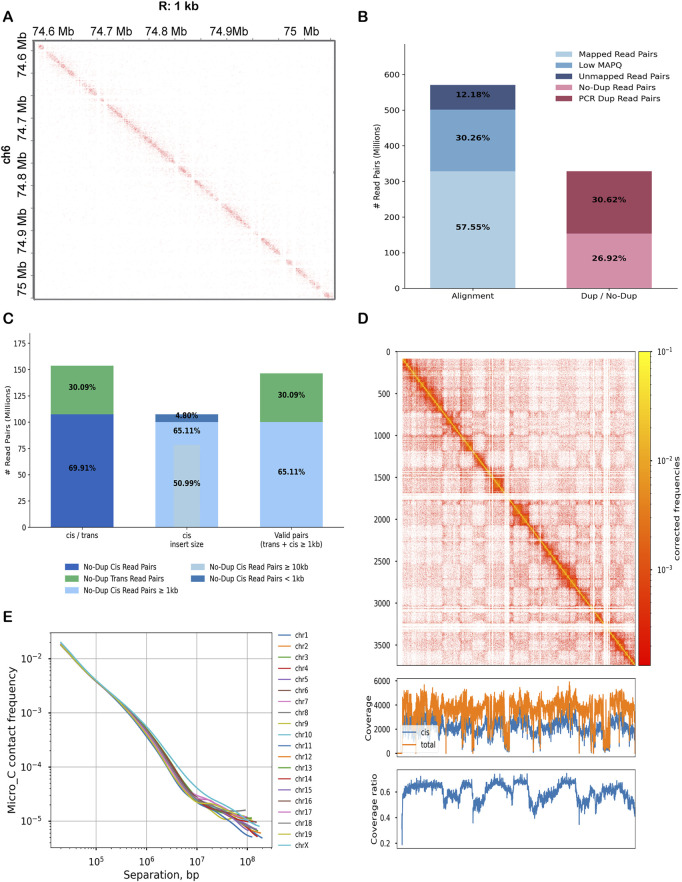
Genome-wide Micro-C contact mapping and quality assessment. **(A)** Chromatin contact map for a representative region of chromosome 6, 1 kb resolution (74.0–75.1 Mb). **(B)** Read the processing summary from total to high-quality contacts. Bars indicate total read pairs, alignment efficiency, and proportions of read pairs retained after duplicate removal and quality filtering. Color coding distinguishes total read pairs, mapped read pairs, low Mapping Quality (MAPQ), unmapped read pairs, no-dup read pairs, and PCR dup read pairs. **(C)** Distribution of valid read pairs by interaction type and genomic distance. From left to right, bars represent: all no-dup read pairs; classification into cis (intra-chromosomal) and trans (inter-chromosomal) contacts; cis contacts further stratified by genomic distance (≥10 kb, ≥1 kb, <1 kb); and valid pairs (trans plus cis ≥1 kb). **(D)** Chromosome-wide contact coverage on chromosome 6. The upper heatmap depicts the high-resolution cis contact map, while the lower line plots show normalized coverage (cis and total) across genomic bins. **(E)** Decay of contact probability with increasing genomic separation, P(s).

We applied Micro-C, a high-resolution chromatin interaction assay, to the postnatal day 0/1 (P0/1) mouse cochlea. This developmental stage represents a critical window for maturation of the auditory system, including sensory hair cells, where precise enhancer-driven gene regulation is essential. Using four total replicates [three biological replicates (Replicate 1, 2, 3) and one technical replicate (Replicate 4)], we aligned paired-end reads to the mm10 genome with BWA-MEM. We then used custom scripts to filter our data to retain only chimeric (distal) read pairs and remove self-ligation and short-range artifacts. Read pairs were defined as chimeric if they mapped to different chromosomes, showed atypical mapping orientation (such as both reads aligning to the same strand), or were separated by more than 2,000 base pairs in the linear genome. These pairs were selected to identify candidate long-range interactions. As shown in Supplementary Figure S1A (unfiltered), dense contact signals include high levels of background noise from self-ligation and short-range artifacts. After filtering for chimeric reads (Figure 1A), the contact map becomes sparser but cleaner, enhancing the visibility of biologically meaningful long-range contacts. Filtered reads were then processed using Dovetail pipeline modules featuring *pairtools*, including parsing, sorting, duplicate removal, and contact classification steps, to generate a high-resolution chromatin contact map. We also excluded reads mapping to mitochondrial and Y chromosomes to eliminate non-nuclear and sex-specific signals. This approach enabled genome-wide classification of interaction types (including cis/trans and short/long-range) with enriched detection of functional enhancer-promoter interactions, analyzed at high resolutions (5–10 kb binning) to capture long-range interactions.

### 2.2 Quality assessment of micro-C library

We next assessed library quality and interaction complexity to ensure this map accurately reflects cochlear chromatin architecture. From approximately 570 million total read pairs, we obtained 57.6% uniquely mapped reads, with 26.9% passing all quality filters, resulting in a high-quality dataset with strong signal and low noise (Figure 1B; Table 1). Interaction classification revealed that 70% were intra-chromosomal (cis), with over 65% spanning ≥1 kb (half exceeding 10 kb), demonstrating the efficiency of Micro-C in capturing long-range interactions (Figure 1C). In total, 95.2% of mapped unique reads met validity criteria (cis ≥1 kb or trans), providing us with a detailed level of resolution of cochlear-specific chromatin loops and domains. Parallel analysis of unfiltered data confirmed our filtered data set was biologically enriched rather than technically biased (Supplementary Figures S1A and S1B; Supplementary Table S1). To show replicate-level resolution of library quality, we generated summary plots for each replicate showing mapping performance, PCR duplication, cis/trans ratios, and interaction distance distributions (Supplementary Figures S2A–S2L; Supplementary Table S2).

**TABLE 1:** Summary of valid Micro-C read pairs and interaction classifications for the filtered dataset. Read pairs were aligned to the reference genome and processed through filtering steps to remove low-mapping-quality reads and PCR-derived duplicates.

Category	Count	Percent	Basis of proportion
Total Read Pairs	570,717,912	100.00%	Proportion of Total Read Pairs
Unmapped Read Pairs	69,526,854	12.18%	
Mapped Read Pairs	328,428,402	57.55%	
PCR Dup Read Pairs	174,768,612	30.62%	
No-Dup Read Pairs	153,659,790	26.92%	
No-Dup Cis Read Pairs	107,423,553	69.91%	Proportion of No-Dup Read Pairs
No-Dup Trans Read Pairs	46,236,237	30.09%	
No-Dup Valid Read Pairs (cis ≥ 1 kb + trans)	146,280,218	95.20%	
No-Dup Cis Read Pairs < 1 kb	7,379,572	4.80%	
No-Dup Cis Read Pairs ≥ 1 kb	100,043,981	65.11%	
No-Dup Cis Read Pairs ≥10 kb	78,354,494	50.99%	

We further assessed library complexity using *preseq* and demonstrated approximately 267 million distinct read pairs from a total of 300 million subsampled reads (Table 2). This high complexity, close to the theoretical limit for our sequencing depth, confirms that our library captured diverse chromatin interactions with minimal technical redundancy.

**TABLE 2:** Library complexity for Micro-C data. Number of distinct reads at increasing sequencing depths, estimated using *preseq*. Columns report the estimated number of distinct reads (EXPECTED_DISTINCT) and the corresponding lower and upper bounds of the 95% confidence interval (LOWER_0.95CI, UPPER_0.95CI).

TOTAL_READS	EXPECTED_DISTINCT	LOWER_0.95Cl	UPPER_0.95CI
0	0	0	0
100000000.0	95974421.5	95970198.2	95978041.8
200000000.0	184700908.0	184692670.9	184708955.2
**300000000.0**	**267136769.0**	**267124233.9**	**267148230.8**
400000000.0	344065773.6	344046637.9	344082223.7
500000000.0	416137741.2	416109873.3	416161402.5
600000000.0	483900359.7	483866067.2	483931801.4
700000000.0	547819469.1	547774217.6	547858847.1
800000000.0	608285724.7	608231622.8	608340919.6
900000000.0	665648432.8	665579552.1	665720222.7
1000000000.0	720199866.2	720106947.0	720288862.8
1100000000.0	772189476.3	772077691.2	772309832.9
1200000000.0	821848055.1	821708030.8	822014761.6
1300000000.0	869371948.7	869168944.0	869595738.2
1,400,000,000.0	914945397.6	914659092.0	915234722.5
1500000000.0	958702188.0	958332023.7	959094825.7
1,600,000,000.0	1000787105.4	1000316868.8	1001316487.7
1700000000.0	1041318770.2	1040721262.9	1042018554.2
1800000000.0	1080423988.1	1079668252.7	1081312396.3
1900000000.0	1118197784.8	1117205353.6	1119297901.5
2000000000.0	1154727768.7	1153397524.6	1156064912.0

To evaluate the consistency and depth of chromatin interaction, we used *cooltools coverage* to analyze contact coverage across the genome. For example, on chromosome 6 (Figure 1D), we observed stable cis and total coverage levels, with only minor local dips, likely reflecting regions of low mappability or structural variation, which is common in these assays. The cis-to-total coverage ratio averaged around 0.6, supporting the predominance of biologically meaningful intra-chromosomal interactions, as shown in our bar plot. To evaluate replicate reproducibility, we computed pairwise Pearson correlations between biological replicates at both 10 kb and 20 kb resolutions using HiCExplorer’s *hicCorrelate* (Supplementary Figures S1D–S1G). Pairwise correlations showed overall consistency among replicates, with clear diagonal clustering at both 10 kb and 20 kb resolutions. This supports reproducibility of the dataset and provides a rationale for using merged contact maps in downstream analyses. In addition, we produced replicate-specific contact heatmaps of chromosome 6 at the same resolutions to visually confirm interaction consistency across samples (Supplementary Figures S3A–S3H).

Finally, we generated a genome-wide P(s) curve to assess the decay of contact frequency with increasing genomic distance (Figure 1E). After smoothing and normalization, all chromosomes exhibit highly similar decay profiles, consistent with polymer physics-based models of chromatin folding (Supplementary Figure S1H). These quality metrics confirm that our Micro-C dataset is robust enough to support high resolution mapping of regulatory interactions, including enhancer-promoter loops and domain-level features specific to cochlear cells.

### 2.3 Genome-wide architecture in cochlear cells

**FIGURE 2:**
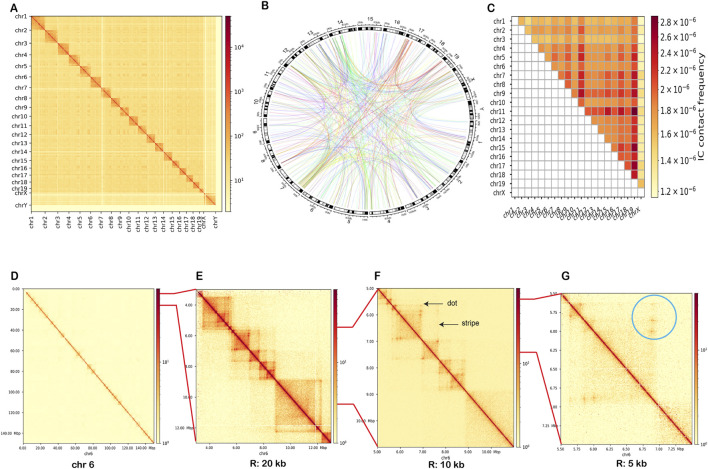
Genome-wide and inter and intra-chromosomal chromatin architecture. **(A)** Genome-wide Micro-C interaction matrix at 10 kb resolution, displaying contact frequencies between all chromosome pairs. **(B)** Circos plot showing the top 500 inter-chromosomal contacts identified by *Fit-Hi-C*, ranked by contact frequency. **(C)** Heatmap summarizing average inter-chromosomal contact intensity across all chromosome pairs. Darker colors indicate higher contact frequency. **(D)** Chromosome-wide interaction map for chromosome 6, showing the full range of cis contacts along the chromosome. **(E)** Zoomed-in region of chromosome 6 (4–12 Mb) at 20 kb resolution. **(F)** At 10 kb resolution, showing finer features including prominent dots (loops) and stripes (elongated interaction patterns). **(G)** At 5 kb resolution, illustrating local interaction structures.

With our quality-optimized Micro-C dataset, we first looked at genome-wide interaction frequencies to assess global chromatin organization. We down-sampled our 10 kb resolution matrix by dividing each chromosome into 100 equal-sized bins using *HiCExplorer* (Wolff et al., 2020), allowing us to compare chromatin architecture across chromosomes in a size-independent manner. We then generated a two-dimensional Micro-C interaction matrix to visualize how both cis and trans (intra- and inter-chromosomal) contacts are distributed across nuclear space (Figure 2A). As expected, the strongest signals appeared along the diagonal, reflecting frequent intra-chromosomal interactions, while inter-chromosomal contacts revealed a global nuclear organization. This trend is further supported in Table 3, where intra-chromosomal interactions dominate across all chromosomes.

**TABLE 3:** Genome-wide distribution of intra- and inter-chromosomal interactions.

Chromosome	Number of intra-chromosomal interactions	Number of inter-chromosomal interactions
chr1	71331	15404
chr2	74,302	15,892
chr3	70,599	15,759
chr4	52,336	16,374
chr5	48689	15,928
chr6	58,495	16,819
chr7	40,924	20,390
chr8	47,990	12,679
chr9	35,324	13,950
chr10	55,759	12,874
chr11	30,894	11,489
chr12	42,611	16,609
chr13	44,747	17,216
chr14	37,942	14,232
chr15	33,913	10,335
chr16	38,851	8919
chr17	28,197	13,779
chr18	32,474	9,027
chr19	12,498	8,839
chrX	14,646	13,600

#### 2.3.1 Inter-chromosomal interaction network

To further investigate inter-chromosomal interactions, we analyzed their spatial organization and potential regulatory relevance in cochlear cells. We performed a genome-wide contact analysis at 10 kb resolution using *Fit-Hi-C* (Kaul et al., 2020), enabling the reliable detection of significant chromatin interactions. We extracted the top 500 inter-chromosomal contacts, ranked by contact frequency, and visualized them in a Circos plot (Figure 2B), which displayed an extensive chromatin interaction network spanning multiple chromosomes. The heatmap in Figure 2C provides a simplified, quantitative summary of average inter-chromosomal contact intensity across chromosome pairs. These patterns may reflect coordinated regulation of gene clusters involved in cochlear function. Together, these data demonstrate that Micro-C captures inter-chromosomal chromatin architecture in cochlear tissue. While these trans interactions provide a view of coordinated activity across chromosomes, most regulatory interactions occur within chromosomes. We therefore next examined intra-chromosomal interaction patterns to gain a better understanding of cochlea-specific regulatory landscapes.

#### 2.3.2 Intra-chromosomal contacts and fine-scale topology

Zooming in from the genome-wide view to individual chromosomes, we focused on intra-chromosomal contacts, which play pivotal roles in gene regulation, DNA replication, and the formation of chromatin domains. We specifically focused on chromosomes 6 (Figure 2D) and 13 as model loci due to their disease relevance (Supplementary Figure S4A). To investigate chromatin folding within these chromosomes, we generated interaction maps at 20 kb, 10 kb, and 5 kb resolutions (Figures 2E–G; Supplementary Figures S4B–S4D). Using these multiple scales provided different perspectives, which allowed us to capture both TADs and enhancer-prompter loops.

##### 2.3.2.1 Chromatin compartments and TAD boundary structure

**FIGURE 3:**
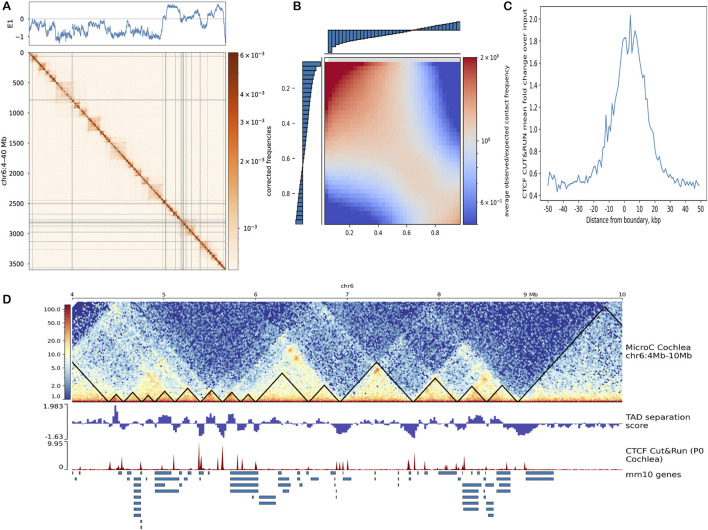
Chromatin compartments and TAD organization. **(A)** Normalized Micro-C contact matrix for chromosome 6 with the first eigenvector (E1) plotted above. Positive E1 values correspond to transcriptionally active A compartments, while negative values indicate inactive B compartments. **(B)** Saddle plot illustrating compartmentalization strength and the matrix shows higher interaction frequencies within the same compartments (A-A and B-B, red) and weaker interactions between compartments (A-B, blue), based on 40 kb binned E1 scores. **(C)** Pileup of CTCF CUT&RUN signal centered on strong TAD boundaries, derived from 10-kb binned insulation profiles. **(D)** Zoomed-in 10 kb resolution contact map of chromosome 6 (4–10 Mb) with TAD boundaries (black triangles). Below the map are TAD separation scores and CTCF binding tracks, alongside annotated genes.

To build on the structural organization observed at the inter- and intra-chromosomal levels, we next explored how higher-order domain architecture is partitioned in cochlear cell chromatin. Using the *cooltools* framework, we analyzed normalized Micro-C contact matrices at 10 kb resolution to assess both chromatin compartments and topologically associating domains (TADs). By performing eigenvector decomposition, we extracted the first principal component (E1), which effectively separated transcriptionally active (A; positive values) and inactive (B; negative values) compartments across the genome. Distinct A/B compartment patterns were clearly observed on chromosomes 6 (Figure 3A; Supplementary Figure S5A) and 13 (Supplementary Figure S5B), where regions within the same compartment showed higher interaction frequencies, forming the expected plaid-like diagonal pattern in the contact maps. To quantify compartmentalization strength, we generated a saddle plot based on E1 scores (Figure 3B). 40 kb genomic bins were ranked by E1 values along both axes to compare interaction frequencies between compartments. Strong intra-compartment interactions appeared in red (upper-left for B-B, lower-right for A-A), while inter-compartment interactions (A-B) were weaker (blue, upper-right and lower-left). A corresponding saddle strength profile (Supplementary Figure S5C) showed that same-compartment interactions were strongest at short distances (1–5 Mb), consistent with the expected decay of compartment strength with increasing genomic distance.

For domain-level analysis, we calculated insulation scores across the genome to identify TAD boundaries, using 10 kb resolution matrices for segmentation. Insulation scores measure how effectively each region separates interactions between its neighboring regions. Regions with low insulation scores, where interaction levels drop, were considered potential boundaries between TADs. Supplementary Figure S5D shows an example region from chromosome 6, where clear insulation scores correspond to the segmentation of the genome into distinct structural units. These insulation profiles validate the segmentation of cochlear chromatin into discrete domains and support downstream analyses of loop formation and enhancer-promoter specificity within these architectural units. To assess the molecular basis of these boundaries, we analyzed them alongside CTCF CUT&RUN data. CTCF is a well-known insulator protein whose binding at specific loci helps to define genomic boundaries. A 1D pileup plot of CTCF signal centered around strong boundaries (Figure 3C) revealed a clear enrichment of CTCF at boundary positions, reinforcing its role in TAD organization.

To define TADs, we mapped the boundaries onto contact matrices for chromosome 6 (5.5–8 Mb), with TADs highlighted in white (Supplementary Figure S5E). The identified domains aligned well with visible structural features in the 10 kb binned contact maps. For further validation, we compared these *cooltools*-based results with those obtained using *HiCExplorer*’s TAD-calling algorithm, with a delta insulation score threshold of 0.05 to define boundaries. As shown in Figure 3D, *HiCExplorer* identified domain boundaries and separation scores that closely matched those from the *cooltools* approach, including consistent alignment with CTCF peaks and visible TAD structures. In particular, stripes and dots become visible at higher resolutions (Figure 2F), adding finer detail to our understanding of domain-internal structure. Stripes appear as linear extensions from the diagonal in the contact map and represent continuous contacts between a single region and multiple surrounding regions. These features often originate from the borders of self-interacting domains and colocalize with transcription start sites. Stripes are enriched for active transcription features, and intersections between two stripes typically form sharp dots, representing loop-like interactions between promoters and enhancers (Hsieh et al., 2020). The presence of both stripes and dots, across multiple loci, alongside TAD boundaries and CTCF enrichment, suggests gene regulation in cochlear cells relies not only on distinct loops but also on extended, stripe-like regulatory interactions. This domain-level organization provides a comprehensive framework for tissue-specific loops.

##### 2.3.2.2 Genome-wide enhancer-promoter interactions

**FIGURE 4:**
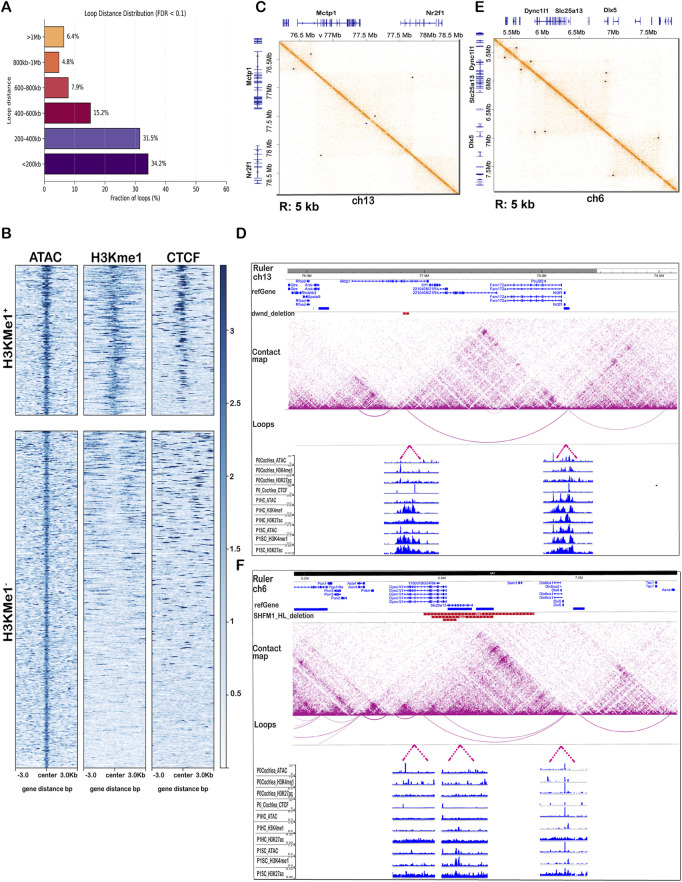
Genome-wide and locus-specific chromatin loops and enhancer-promoter interactions. **(A)** Distribution of chromatin loop distances detected by *Mustache* at 10 kb resolution. Loop distances were grouped into six categories: <200 kb, 200–400 kb, 400–600 kb, 600–800 kb, 800 kb-1 Mb, and >1 Mb. **(B)** Heatmaps of ATAC-seq, H3K4me1, and CTCF signals centered on Mustache-detected loop anchors for cochlea, separated into H3K4me1^+^ (top) and H3K4me1^-^ (bottom) groups. **(C)** Micro-C contact map of chr13 in cochlear cells visualized in Juicebox (10 kb) with identified loops (blue dots) showing a loop between *Mctp1* and *Nr2f1*. **(D)** WashU Epigenome Browser view of the region (10 kb resolution) showing arcs (loops) and *dwnd* deletion (red track) overlapping the contact with integrated P0/1 cochlear ATAC-seq, H3K4me1, H3K27ac, and CTCF tracks, as well as P1HC and P1SC signals for ATAC-seq, H3K4me1, and H3K27ac. **(E)** Micro-C contact map on chromosome 6, highlighting two long-range loops (blue dots) connecting *Dync1i1* with the *Dlx5/6* gene cluster visualized in Juicebox (10 kb resolution). **(F)** WashU Epigenome Browser view of the same region (10 kb resolution) with arcs marking loops and overlapping SHFM1-associated hearing loss deletions (red tracks) with integrated epigenomic signals.

To identify chromatin loops in cochlear cells, we applied the *Mustache* algorithm (Roayaei Ardakany et al., 2020) at both 5 kb and 10 kb resolutions, using a p-value threshold of 0.1, which balances sensitivity and specificity at coarser resolution. At 10 kb resolution, we detected 6,798 chromatin loops (Supplementary Table S3), while the 5 kb resolution analysis identified 3,549 loops. To evaluate the distribution of loop distances, we categorized the genomic distances of each loop into six groups: <200 kb, 200–400 kb, 400–600 kb, 600–800 kb, 800 kb-1 Mb, and >1 Mb (Lee et al., 2022). We next examined how loop detection varied with statistical stringency. Using FDR <0.1, 6,798 loops were detected, whereas applying the more stringent FDR <0.05 threshold yielded 5,356 loops (Supplementary Table S4). The majority of loops are within short- to mid-range distances, with nearly 66% spanning less than 400 kb (Figure 4A). This distribution aligns with the expected frequency of enhancer-promoter interactions, highlighting the sensitivity of our Micro-C data in capturing high-resolution chromatin loops in cochlear tissue.

To investigate the chromatin landscape of cochlea-specific chromatin loops, we used *Mustache*-detected loop anchors and intersected them with whole-cochlea ATAC-seq peaks to define accessible loop anchor regions (n = 756). Heatmaps of ATAC-seq, H3K4me1, and CTCF signals (from cochlear CUT&RUN) at these anchor regions revealed distinct chromatin profiles (Figure 4B). Among these accessible loop anchors, 233 (∼30.8%) were also enriched for H3K4me1, a histone modification marking active and permissive distal enhancers (Barral and Déjardin, 2023) as well as binding signals for CTCF, one key component of long-range chromatin looping (Splinter et al., 2006). In contrast, H3K4me1^-^ anchors showed reduced accessibility and a weaker H3K4me1 signal, with CTCF signals appearing more diffuse. These data demonstrate that, within the cochlea, roughly one-third of accessible loop anchors are associated with enhancer-like chromatin elements.

Extending this to specific cochlear cell types, we intersected ATAC-seq peak data obtained from either purified hair cells (*Atoh1*-GFP^+^ HC) or supporting cells (*Lfng*-GFP^+^ SC) with loop anchors, yielding 709 accessible anchors in HCs and 608 accessible anchors in SCs. Heatmaps of ATAC, H3K4me3 [marker for active and poised promoters (Bernstein et al., 2006)], and H3K4me1 signals highlighted cell type-specific chromatin states (Supplementary Figures S6A and S6B). In HC, 258 of 709 anchors (36.4%) were H3K4me1^+^, 171 (24.1%) were H3K4me3^+^, and 96 (13.5%) carried both marks. In SC, 390 of 608 anchors (64.1%) were H3K4me1^+^, 315 (51.8%) were H3K4me3^+^, and 235 (38.7%) carried both marks. Anchors lacking both H3K4me1 and H3K4me3 exhibited lower accessibility, consistent with the role of H3K4me1 and H3K4me3 in maintaining the open status of chromatin (Wang and Helin, 2025).

##### 2.3.2.3 Locus-level loop analysis

Building on the genome-wide analyses, we next zoomed in on locus-level analysis to connect chromatin architecture with functional biological relevance. We focused on two cochlea disease-linked regions, *NR2F1* and *DLX5/6*, because disease-associated structural variants overlap their loop anchors, providing an opportunity to examine how disruptions at these sites could reshape chromatin organization and impair gene regulation in hearing loss.

###### 2.3.2.3.1 Chromosome 13: *Nr2f1–Mctp1* regulatory locus

To investigate cochlear-specific regulatory interactions at the *Nr2f1* locus implicated in Bosch-Boonstra-Schaaf optic atrophy syndrome (BBSOAS) and the accompanying hearing loss phenotype, we visualized chromatin contact maps at 10 kb resolution on chromosome 13. A prominent long-range interaction was observed between *Mctp1* and the *Nr2f1* loci (Supplementary Figures S2A–S2D), identified using *Mustache* loop calls (Roayaei Ardakany et al., 2020) represented as blue dots in Juicebox (Figure 4C; Table 4; Supplementary Table S3) (Durand et al., 2016), and as arcs in the WashU Epigenome Browser (Li et al., 2019) aligned with epigenomic signal tracks (Figure 4D). The observed/expected (O/E) ratio measures how much stronger a contact is than expected by chance, calculated with *Fit-Hi-C* at 10 kb resolution, and values above 50 (merge data set) indicate unusually strong long-range interactions (Table 4; Supplementary Table S3). This clear arc connecting *Mctp1* and *Nr2f1* reveals a strong, long-range interaction between the two regions. Notably, both *Mctp1*and *Nr2f1* loci are located within the same TAD, providing additional evidence of a potential enhancer-promoter regulatory relationship.

**TABLE 4:** Quantitative loop strength at disease-associated loci for each replicate (O/E, observed/expected contact enrichment (*Fit-Hi-C*, 10-kb); p, binomial P-value; q, FDR-adjusted P-value).

Loop	Replicate	O/E ratio	p-value	q-value
Nr2f1–Mctp1	Rep1	42.52	2.32 × 10^−2^	1.00
	Rep2	—	—	—
	Rep3	55.20	1.80 × 10^−2^	1.00
	Rep4	53.45	6.00 × 10^−8^	5.56 × 10^−4^
	Merged	55.16	4.27 × 10^−12^	6.85 × 10^−8^
Dync1i1–Dlx5/6 (Loop 1)	Rep1	29.07	3.38 × 10^−2^	1.00
	Rep2	—	—	—
	Rep3	—	—	—
	Rep4	49.42	2.00 × 10^−10^	3.52 × 10^−6^
	Merged	31.90	4.02 × 10^−9^	4.12 × 10^−5^
Dync1i1–Dlx5/6 (Loop 2)	Rep1	46.54	8.98 × 10^−4^	1.00
	Rep2	—	—	—
	Rep3	33.22	2.96 × 10^−2^	1.00
	Rep4	72.36	3.00 × 10^−8^	1.00 × 10^−8^
	Merged	53.91	2.73 × 10^−21^	1.02 × 10^−16^

To assess regulatory activity at the loop anchors, we overlaid cochlear ATAC-seq, H3K4me1, H3K27ac, and CTCF signal tracks (P0/1 cochlea), as well as ATAC-seq, H3K4me1, and H3K27ac data from HC and SC. These tracks showed strong chromatin accessibility and enhancer-associated histone marks at both ends of the loop, supporting the presence of a functional enhancer-promoter interaction. In particular, strong H3K27ac and H3K4me1 signals were observed at the *Nr2f1* promoter in both P1 HC and SC, consistent with active gene expression in both cochlear cell types (Supplementary Figure S6C) (Orvis et al., 2021). Importantly, a hearing loss-associated deletion in mice (Tarchini et al., 2018), shown as a red track, overlaps with the distal loop anchor near *Mctp1*. This overlap suggests that disruption of this enhancer region may interfere with *NR2F1* regulation and contribute to the observed auditory phenotype.

In contrast, we examined the same genomic region using the ENCODE human data for other non-cochlear cell types [ENCSR228TUX, human differentiated motor neuron cells (Zhang et al., 2022); and ENCSR968KAY, GM12878 cells (Rao et al., 2014)]. Neither of these cell types exhibited strong or specific long-range interactions between *Mctp1* and *Nr2f1* (Supplementary Figures S6D and S6E), suggesting this interaction is cochlea-specific. These findings provide evidence for a tissue-specific chromatin loop critical for auditory gene regulation, reinforcing the role of non-coding disruptions in the *NR2F1* regulatory landscape in hearing loss.

###### 2.3.2.3.2 Chromosome 6: *Dync1i1–Dlx5/6* regulatory locus

We next examined the unbiased chromatin structure at the *Dync1i1-Dlx5/6* locus on chromosome 6, which is implicated in Split-Hand/Foot Malformation Type 1 (SHFM1) and associated with hearing loss. At 10 kb resolution, we visualized this region and identified two distinct long-range loops between *Dync1i1* and the *Dlx5/6* gene cluster using Juicebox with loop calls, marked by blue dots (Figure 4E; also see Figures 2E–G and Table 4; Supplementary Table S3). Both loops show strong enrichment (O/E ∼32 and ∼54 in the merged dataset), confirming that these long-range contacts are quantitatively robust. A visible stripe extending from a neighboring region suggests ongoing loop extrusion activity, indicative of dynamic regulatory interactions. These features indicate a structurally complex regulatory environment involving *Dync1i1-Dlx5/6* and surrounding loci. To further confirm this interaction, we integrated our contact maps into the WashU Epigenome Browser again alongside a custom bed file marking known SHFM1-associated hearing loss deletions (Figure 4F). The red SHFM-HL tracks overlap with two prominent chromatin loops, reinforcing the hypothesis that these enhancer regions physically interact with the *Dlx5/6* locus in cochlear chromatin and that their disruption may impact chromatin architecture in a region linked to hearing loss.

Next, we cross-examined epigenetic and transcriptomic data to interpret the function of those interactions. We observed ATAC-seq peaks at loop anchors in both cell types, indicating accessible chromatin, with H3K4me1 enriched across the locus, particularly in supporting cells. In contrast, the H3K27ac signal was low in both hair cells and supporting cells. These epigenetic configurations suggest that those elements are not actively engaged in transcription in the cochlea, consistent with the low expression of *Dlx5* in cochlear cells (Orvis et al., 2021). Interestingly, *Dlx5* is highly expressed in vestibular supporting cells (Supplementary Figure S6F) (Orvis et al., 2021), suggesting that the chromatin architecture at this locus is preserved between vestibular and cochlear organs of the inner ear. To assess chromatin interaction differences across tissues, we examined the same genomic region using the ENCODE datasets (Supplementary Figures S6G and S6H). While weak or diffuse interactions were present in these non-cochlear samples, the loops lacked the defined architecture and intensity observed in our dataset. These comparisons suggest that while basal-level interactions may exist across tissues, loop strength and organization at *Dync1i1-Dlx5/6* are selectively enhanced in the cochlear chromatin. Together, these findings support that the *Dync1i1*-*Dlx5/6* regulatory domain is organized through enriched multi-loop interactions observable in cochlear chromatin, and that SHFM1-associated deletions disrupt this architecture, potentially contributing to gene misregulation.

### 2.4 Hair cell promoter-enhancer interactions

Although our Micro-C data comes from whole cochlear tissue containing many diverse cell types in the sensory epithelium, we can use additional bioinformatic tailoring strategies to narrow our focus to specific cell types of interest, such as hair cells or supporting cell subtypes. To functionally interpret enhancer-promoter interactions relevant to hair cells, we focused on interacting regions overlapping with a subset of hair cell-specific genes. The low abundance of hair cells in bulk tissue limits the direct resolution of hair cell-specific loops in our Micro-C data, but by focusing on chimeric fragments anchored at hair cell gene promoters, we can infer likely promoter-enhancer interactions linked with hair cell gene regulation.

**FIGURE 5:**
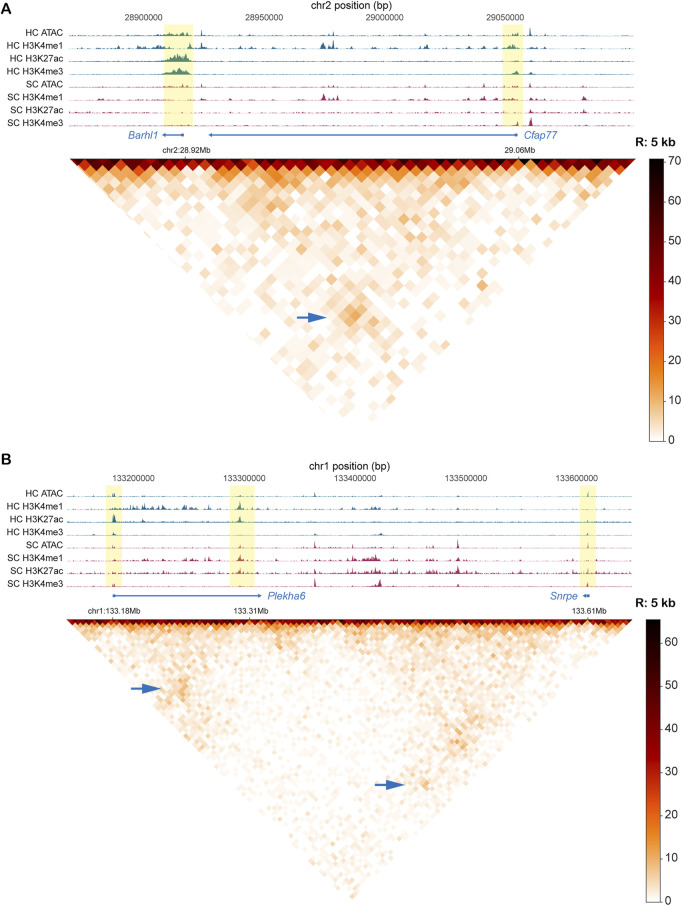
Interactions between known hair cell gene promoters and inferred enhancer regions in Micro-C data and *Fit-Hi-C* analysis. **(A)** Interaction between regions containing promoters of two hair cell genes (*Barhl1* and *Cfap77).* Heatmap is binned at 5 kb. **(B)** Enhancer interaction with *Plekha6* promoter (hair cell gene) and *Snrpe* promoter (expressed in both hair cells and supporting cells). Heatmap is binned at 5 kb. For both A and B, tracks include bigwig signal files for ATAC and various histone modification CUT&RUN. All tracks represent purified P1mouse hair cells. Yellow highlighted bars identify the interacting regions in the signal tracks. Signal strength in the heatmap is depicted by contact frequency (darker signals = more interactions between loci). Blue arrows identify the intersection of two interacting loci.

For this analysis, we extracted hair cell-specific information using a published single-cell multiomic dataset from the P1 mouse cochlea (Iyer et al., 2022), which includes paired RNA-seq and ATAC-seq data from individual cells. We used *Seurat* (Hao et al., 2024) to determine differentially expressed genes (DEGs) of hair cells compared to the remaining sensory epithelium (SE) (Supplementary Figures S7A and S7B). Then, we obtained approximate promoter region coordinates for these DEGs, and used these to filter our Micro-C data after the *Fit-Hi-C* step. We examined the *Fit-Hi-C* metrics to pinpoint the most significant and highest occurring interactions with these genes. We cross-checked our observations by looking at various bulk chromatin accessibility and epigenetic datasets for both purified hair cells and supporting cells to validate which interactions are indeed most likely enriched in hair cells compared to other SE cell types. Despite the outnumbering of other SE cell types to hair cells, we were able to visualize some strong interactions between hair cell gene promoters and distal elements in our Micro-C dataset by heatmaps at various resolutions that were also supported by chromatin accessibility and histone modification patterns. Across multiple bins, we show strong signals which we can infer are hair cell-specific interactions. We found examples of two distal interacting regions around the sites of the promoter regions of hair cell genes *Barhl1* and *Cfap77* (Figure 5A). Enriched signals for both H3K4me1 and H3K4me3 at both loci indicate enhancer-promoter interplay between these two regions. We note several strong examples of the complex staple behaviors of enhancers (or other distal elements), including the regulation of a single region by multiple enhancers, as well as single enhancers regulating multiple loci. For example, we observed a putative enhancer interacting with both the *Plekha6* and *Snrpe* promoters. In this region, both promoters are located near loop anchors, and each shows distinct epigenomic configurations. The *Plekha6* promoter displays high H3K27ac and H3K4me3 signals specifically in hair cells, consistent with its expression being enriched in hair cells. Although we do not have chromatin interaction data from purified hair cells or supporting cells, the strong correlation between the epigenomic profile and transcriptomic data supports the hypothesis that this enhancer actively regulates *Plekha6* in hair cells. In contrast, the *Snrpe* promoter shows a similar level of chromatin accessibility and active H3K27ac marks in both hair cells and supporting cells, matching its comparable expression in both cell types. This suggests that the same enhancer may contribute to different regulatory outcomes depending on promoter-specific conditions (Figure 5B). Additionally, the *Bdnf* gene appears to be regulated by multiple distal upstream enhancers (Supplementary Figure S7C, 25 kb). Other enhancer-promoter interactions that are likely hair cell-specific were visualized for *Msra*, *Mgat5b*, *St8sia2*, *Efcab6*, *B3gnt4*, and several others (Supplementary Figure S6A). We were also able to point out some likely relationships specific to outer and inner hair cell subtypes, reflected by heatmaps of OHC-specific gene *Mslnl* and IHC-specific gene *Fgf8* (Supplementary Figure S8A). Our gene expression analysis of the single-cell multiome dataset further reflects the strict hair cell specificity of these genes (Supplementary Figures S8B and S8C). Thus, this DEG-centric filtering of our data offers us a glimpse into the potential distal relationships most relevant to hair cells.

Although some of the interactions we identified do not necessarily appear strong by heatmap or *Fit-Hi-C* metrics, likely due to hair cells being underrepresented in bulk tissue, we still detected known enhancer-promoter relationships for genes such as *Atoh1* and *Rasd2*. These were supported by their epigenetic configurations, suggesting biological relevance despite low contact counts (Supplementary Table S5; Supplementary Figure S9). To facilitate interpretation, Supplementary Table S5 also includes an additional column annotating known deafness-associated genes, enabling readers to directly identify promoter-enhancer interactions that may be functionally relevant to hearing loss. This highlights the sensitivity of our approach and its potential to identify hair cell-specific regulatory interactions. It further indicates that these bioinformatic adaptations can be used to focus on any cell type of interest with a known gene signature profile to unlock insights into its distal regulome.

## 3 Discussion

In this study, we generated the high-resolution chromatin interaction map of the neonatal mouse cochlea using Micro-C, providing a deeper understanding of how genes are organized and regulated at the transcriptional level in the inner ear. By using Micro-C technology, we overcame the limitations of conventional 3C-based methods and successfully captured chromatin interactions. This allowed us to identify enhancer-promoter loops at specific loci related to hereditary hearing disorders, offering a structural explanation of how non-coding genomic mutations may contribute to hearing loss.

One of the central challenges in gene regulation is identifying which distal regulatory elements interact with which promoters, especially in complex tissues like the inner ear. Enhancers can act over large genomic distances and often skip nearby genes, making linear genome annotations unreliable predictors of regulatory relationships (Miele and Dekker, 2008; Lieberman-Aiden et al., 2009). To address this, we applied Micro-C, a technique capable of nucleosome-level resolution, and analyzed the resulting contact maps at 5–10 kb resolution. This enabled us to define enhancer-promoter loops directly, without relying solely on epigenomic marks or linear proximity. By integrating these interaction maps with histone modification profiles, chromatin accessibility data, and transcriptional activity from both bulk and single-cell datasets, we systematically linked distal regulatory regions to putative gene targets. ATAC-seq, H3K4me1, and CTCF signals centered on loop anchors further reinforced their regulatory potential (Figure 4B; Supplementary Figures S6A and S6B). We used this strategy at multiple scales, from genome-wide classification of enhancer-enriched loop anchors to focused analyses of disease-relevant loci like *Nr2f1* and *Dlx5/6*, and ultimately to cell-type-refined interactions using single-cell multiomic filtering.

One of our examples was the identification of a long-range chromatin loop between *Mctp1* and the *Nr2f1,* a regulatory interaction previously suggested in mouse models but never mapped at high resolution in the cochlea. BBSOAS (OMIM 615722) is a rare neurodevelopmental disorder caused by *NR2F1* (nuclear receptor subfamily 2, group F, member 1) haploinsufficiency (Brown et al., 2009; Martín-Hernández et al., 2018). This condition is characterized by optic atrophy, intellectual disability, hypotonia, autism spectrum disorder, and hearing impairment, the latter of these affecting approximately 20% of individuals with this disorder (Chen et al., 2016). *NR2F1* encodes COUP-TF1 (Chicken Ovalbumin Upstream Promoter-Transcription Factor 1 protein), a transcription factor essential for neuronal differentiation and sensory organ development. During embryogenesis, *NR2F1* is expressed early in the development of the otic vesicle and later in the development of the cochlear duct and maturation of the organ of Corti (Brown et al., 2009; Tarchini et al., 2018). Genetic changes associated with BBSOAS include point mutations in the DNA-binding domain (DBD) or ligand-binding domain (LBD), frameshifting and non-frameshifting insertions/deletions, whole or partial gene deletions, and chromosomal rearrangements (Brown et al., 2009; Chen et al., 2016; Martín-Hernández et al., 2018). The severity of auditory impairment varies based on the specific type of mutation. Patients with *NR2F1* microdeletions (400–500 kb at or near both breakpoints) on the 5q15 region often exhibit severe congenital sensorineural hearing loss and elevated ABR thresholds, especially at lower frequencies, despite having a structurally normal cochlea (Brown et al., 2009). Similarly, homozygous *Nr2f1-*null mice show cochlear hypoplasia (with 1–1.25 turns instead of the typical 2.5), malformed semicircular canals, and auditory nerve dysfunction (Tang et al., 2006). These structural defects are accompanied by disrupted Notch signaling, abnormal cochlear patterning, increased sensory epithelial cell proliferation, and extra inner and outer hair cells, particularly in the mid-to-apical region of the cochlea (Tang et al., 2006). In contrast, heterozygous *Nr2f1* ^*+/−*^ mice (analogous to human BBSOAS patients) do not show structural abnormalities in the organ of Corti. They do, however, exhibit ABR threshold shifts, particularly at low frequencies (4–16 kHz), and no significant change in the DPOAE threshold, a pattern also observed in BBSOAS patients (Chen et al., 2016; 2020). Some heterozygous mice also demonstrate mild ataxia and vestibular dysfunction, mirroring the balance issues and hypotonia reported in individuals with BBSOAS (Chen et al., 2020).

In the mouse cochlea, one element in the *Mctp1* gene (multiple C2 domain and transmembrane region protein 1) acts as a distal enhancer of *Nr2f1* (Tarchini et al., 2018). The deaf wanderer (*dwnd*) mouse model carries a 53 kb deletion within the *Mctp1* gene, removing exons 11–15 and nearby intronic sequences. This deletion occurs at the 76.8 Mb position on mouse Chromosome 13, which is 1.4 Mb away from the *Nr2f1* gene at the 78.2 Mb position (Tarchini et al., 2018). Despite leaving the *Nr2f1* coding sequence intact, this deletion disrupts a long-range enhancer element, resulting in a 50% reduction in its expression in the cochlea, while expression in the retina remains unaffected (Tarchini et al., 2018). Homozygous *Mctp1* ^*dwnd/dwnd*^ mice exhibit moderate-to-severe hearing loss (elevated ABR thresholds of 25–45 dB across multiple frequencies) and similar morphological changes in cochlear and vestibular systems as homozygous *Nr2f1*-null mice, contributing to phenotypes of circling behavior and balance dysfunction (Tarchini et al., 2018). *Mctp1* ^*+/dwnd*^ *Nr2f1* ^*+/−*^ mice show the same cochlear and vestibular defects seen in *Nr2f1*-null mice, confirming that the *dwnd* deletion impairs *Nr2f1* expression, not *Mctp1* function directly. This is also supported by the normal development, hearing ability, and intact cochlear and vestibular structures seen in *Mctp1* knockout mice (Tarchini et al., 2018; Chen et al., 2020). These results show that the deletion of *Mctp1* ^*dwnd*^ disrupts a critical enhancer of *Nr2f1*, which leads to hearing loss and balance issues due to abnormal inner ear development and compromised Notch signaling. Furthermore, decreased expression of *Nr2f1* causes axonal misguidance, underlining its role in the auditory system in both mice and humans. At the chromatin level, we observed a clear arc connecting *Mctp1* and *Nr2f1*, located within the same TAD (Figures 4C,D, 10 kb resolution), indicating a stable long-range regulatory interaction between these loci, further supported by a high observed/expected enrichment (O/E = 55.1; q = 6.9 × 10^−8^) consistent with the robustness of this interaction (Table 4; Supplementary Table S3). This loop is supported by strong chromatin accessibility and enhancer-associated histone marks (H3K27ac and H3K4me1) at both anchors. In both hair cells and supporting cells, the *Nr2f1* promoter exhibits an active chromatin profile, consistent with its transcriptional activity in the cochlea. The overlap of a long-range chromatin loop between the *Mctp1* and the *Nr2f1* with a known hearing loss-associated deletion (*dwnd_*deletion) provides evidence that structural variants can disrupt enhancer-promoter interaction, leading to *Nr2f1* dysregulation, even in the absence of coding mutations. This observation is consistent with clinical findings of BBSOAS, where *NR2F1* haploinsufficiency causes sensorineural hearing loss, and underscores the importance of considering non-coding regions in genetic diagnoses.

Similarly, our analysis of the *Dync1i1-Dlx5/6* locus revealed chromatin loops that overlap with deletions linked to SHFM1 (OMIM #220600) and associated hearing loss. It is a congenital limb disorder characterized by the absence or malformation of central fingers or toes, often resulting in cleft-like structures in the hands and feet (Bademci et al., 2020). While some cases involve isolated limb anomalies, it is frequently associated with craniofacial malformations, intellectual disability, and sensorineural hearing loss, affecting 35% of patients (Allen et al., 2014). The SHFM1 locus is located on chromosome 7q21.3 and contains six protein-coding genes: *DYNC1I1*, *SLC25A13*, *C7orf76*, *SHFM1* (*DSS1*), and *DLX6* and *DLX5* (Tayebi et al., 2014). The primary cause of SHFM1 is the altered expression of distal-less homeobox 5 (*DLX5*) and *DLX6*, which are critical for limb development by regulating the apical ectodermal ridge (AER), the key signaling center for limb outgrowth (Birnbaum et al., 2012a). However, many SHFM1 cases lack coding mutations in *DLX5/6*, suggesting that changes in long-range interactions of regulatory elements controlling their expression are responsible for the disorder (Birnbaum et al., 2012b). The *DYNC1I1* gene encodes a subunit of the cytoplasmic dynein motor protein complex, which is primarily involved in retrograde axonal transport in neurons. Although *DYNC1I1* does not directly contribute to limb development, exons 15 and 17, located approximately 900 kb from *DLX5/6*, function as enhancers. These enhancers are known to regulate *DLX5/6* expression through chromatin looping, both in the limbs and the otic vesicle (inner ear) in both mice and humans (Brown et al., 2010).

Most SHFM1 cases involve deletions or chromosomal rearrangements that remove or physically separate these enhancers from their target genes, leading to *DLX5/6* misregulation during embryonic development in the limb, branchial arch, inner ear, and forebrain in zebrafish, mice, and humans. Genomic studies in SHFM1 patients have shown that the severity of hearing impairment correlates with the location and size of the chromosomal breakpoint within the 7q21.3 region, which determines how much of the *DLX5/6* regulatory landscape is affected. For example, a patient with SHFM1 and sensorineural hearing loss (SNHL) was found to carry a 6.3 Mb deletion at 7q21.13-q21.3 (chr7: 89,993,838–96,278,971, hg19), which did not remove *DLX5/6* but removed *DYNC1I1* exons 15 and 17 along with other genes (Ambrosetti et al., 2023). Another case involved a 5,115 bp deletion at 7q21.3, located 65 kb from *DLX6* and 80 kb from *DLX5*, which disrupted an inner ear-specific enhancer. Despite leaving *DLX5/6* structurally intact, this deletion led to hearing loss and cochlear malformations (Brown et al., 2010). Further supporting this mechanism, a pericentric inversion (chr7: 29,043,157–96,185,954) displaced *DYNC1I1* exons 15 and 17 nearly 67 Mb away from *DLX5/6*, preventing proper enhancer-promoter interactions and leading to SHFM1 and hearing loss (Birnbaum et al., 2012b). In mouse models, deletion of these exons caused *Dlx5/6* misregulation and cochlear defects, mirroring the human phenotype (Birnbaum et al., 2012a; Tayebi et al., 2014). *Dlx5/6* knockout mice have severe inner ear malformations, suggesting that *DLX5/6* function is essential for auditory system development (Brown et al., 2010). In human-derived lymphoblastoid cell lines from patients with *DYNC1I1* enhancer deletions, *DLX5/6* expression was reduced to approximately 40%–45% of normal levels (Ambrosetti et al., 2023). 3C and DNA FISH analyses have shown that in normal tissue, exons 15 and 17 of *DYNC1I1* physically interact with the *DLX5/6* promoter. However, in SHFM1 patients with enhancer deletions or inversions, these critical interactions are disrupted, leading to gene misregulation (Birnbaum et al., 2012a). When we examined the same genomic region in human motor neuron-derived cells and GM12878 lymphoblastoid cells, we observed loop formation across the *DYNC1I1-DLX5/6* region (Supplementary Figures S6G and S6H). However, the interaction patterns differed, with weaker connectivity at the specific anchor sites that showed a stronger signal in cochlear tissue, suggesting that the regulatory looping architecture at this locus is selectively reinforced in the cochlea (Figure 4E, 10 kb resolution). Interestingly, not all SHFM1 patients with *DYNC1I1* deletions experience hearing loss. This variability displays that other enhancer elements, such as those located in *SLC25A13* (e.g., *hs1642*), may also regulate *DLX5/6* expression in the cochlea (Ramos-Zaldívar et al., 2016). Although some mice that are homozygous for the *Slc25a13* deletion are deaf, the response in humans is more variable; some patients present with hearing loss, while others do not (Johnson et al., 2018).

Our results align with findings from a mouse model carrying the *hspn* deletion (Johnson et al., 2018), which shows hyperactive circling behavior and inner ear defects. The refined *hspn* candidate region included part of *Dync1i1* (Chr 6: 5.73–6.22 Mb) and all of *Slc25a13* (Chr 6: 6.04–6.22 Mb) and *Sem1* (Chr 6: 6.56–6.58 Mb). They detected a large deletion in the *Slc25a13* gene, but no DNA alterations were found in exonic regions of *Dync1i1* or *Sem1*. While the previous work emphasized the *Slc25a13* deletion, it is important to note that this gene sits immediately downstream of *Dync1i1.* This suggests that both human and mouse data may be describing the same regulatory mechanism, but from different genomic angles, either by directly disrupting *Slc25a13* or by compromising regulatory elements embedded within *Dync1i1*. We observed two separate loops in our data, one spanning chr6:5,854,999–6,894,999 within *Dync1i1*, and another spanning chr6:6,004,999–6,884,999 overlapping both *Dync1i1* and *Slc25a13*. This supports the complex chromatin architecture, including a prominent stripe extending from a neighboring region into the *Dlx5/6* locus, which suggests ongoing loop extrusion and a dynamic, modular regulatory structure, with both loops also showing strong quantitative support (loop 1 O/E = 31.9, q = 4.1 × 10^−5^; loop 2 O/E = 53.9, q = 1.0 × 10^−16^), reinforcing their biological relevance (Table 4; Supplementary Table S3). In Figure 4F, these two distinct loops are overlaid with a custom bed track marking SHFM1-associated deletions linked to hearing loss (Kouwenhoven et al., 2010; Allen et al., 2014; Tayebi et al., 2014). This data fortifies the hypothesis that these enhancer regions physically interact with the *DLX5/6* locus and that their disruption may alter transcriptional regulation critical for auditory development. Overall, this underlines that *DLX5/6* regulation in the inner ear depends on multiple enhancer elements (Johnson et al., 2018), which may explain the variability in hearing loss severity among SHFM1 patients. Worth noting, we also identified a long-range loop at another hearing loss associated gene locus, the paralogous *Dlx1/2* locus on chromosome 2 (chr2:71, 254, 999–71,545,001), which resembles the chromatin architecture at *Dlx5/6* and is consistent with the hypothesis that *Dlx* gene clusters originate from duplication of an ancestral *Dlx* locus (Ghanem et al., 2003).

Our findings emphasize the importance of mapping non-coding regulatory elements in tissues affected by genetic disorders. Enhancer disruptions are increasingly recognized as pathogenic drivers, but are difficult to interpret without detailed chromatin interaction data. This high-resolution view allowed us to identify enhancer-promoter interactions at critical loci, including *NR2F1* and *DLX5/6*, both of which are linked to hearing loss. Importantly, these interactions overlapped known structural variants associated with human hearing disease, supporting a mechanistic link between non-coding disruptions and transcriptional dysregulation in the auditory system. By showing how distal enhancers physically connect to hearing-related genes within the cochlea, our Micro-C dataset fills a major gap in the study of hereditary hearing loss. This map provides a base for reinterpreting non-coding variants identified in human genetic studies and for the selection of candidate regulatory regions for functional analysis. Beyond *NR2F1* and *DLX5/6*, our map offers a general platform for investigating enhancer-driven mechanisms in hearing loss. The majority of chromatin loops were 200 kb, typical enhancer-promoter distances, and many were anchored at regions marked by open chromatin and active histone modifications. This serves as a rationale for the use of this dataset to determine candidate enhancers for functional validation, particularly in cases where clinical studies implicate non-coding regions. We also detected inter-chromosomal interactions, though they were less frequent than intra-chromosomal ones. These may point to higher-order coordination of auditory gene networks and represent another area for future investigation.

Worth noting, functional enrichment analysis using the GREAT tool (McLean et al., 2010) with top-scoring intra-chromosomal interacting regions showed enrichment of sensory perception related terms, such as sensory perception of chemical stimulus, sensory perception of smell, and detection of stimulus, suggesting possible similarities in chromatin configuration between cells from different sensory organs (Supplementary Table S6). Further investigation to compare chromatin structures between different sensory organs will likely reveal shared regulatory mechanisms.

Our chromatin contact map of the cochlea provides a high-resolution view of enhancer-promoter architecture in this sensory tissue. While we did not isolate single cell types specifically, such as supporting cells or hair cells, the use of whole cochlea preserved important biological signals and regulatory landscapes, including those critical for hair cell function. However, a key limitation is that interactions are derived from bulk tissue, where non-sensory epithelial cells outnumber hair cells and supporting cells. As such, our ability to confidently assign enhancer-promoter loops to sensory epithelial cells is constrained. While we incorporated single-cell multiomic data to filter for interactions linked to hair cell gene expression, this method infers cell-type relevance indirectly and cannot resolve looping patterns exclusive to individual cell populations. Future studies using purified populations or single-cell chromatin conformation technologies will be necessary to definitively resolve enhancer-promoter architecture in rare cochlear cell types such as hair cells. Importantly, we still observed known enhancer-promoter interactions, such as *Atoh1* and *Rasd2*, despite low signal in bulk data, highlighting the sensitivity of our approach and its potential to reveal novel regulatory relationships when applied to purified datasets.

In conclusion, this study establishes a high-resolution chromatin interaction map of the cochlea and links disease-associated non-coding variants to 3D genome organization. By bridging structural variation with enhancer-promoter disruption, this work provides a mechanistic explanation for how non-coding mutations could contribute to hearing loss, even in the absence of exonic mutations. Our dataset serves as a foundational resource for future studies of inner ear gene regulation and opens new directions for studying the role of spatial genome organization in hereditary hearing disorders.

## 4 Methods

### 4.1 Micro-C experiment

Approximately 6 × 10^5^ cells from P0/P1 mouse cochleae were harvested and crosslinked at room temperature using disuccinimidyl glutarate (DSG), followed by formaldehyde, according to the manufacturer’s protocol (Dovetail Micro-C Kit, Cantata Bio, Scotts Valley, CA, United States). Crosslinked cells were permeabilized and digested with micrococcal nuclease to obtain predominantly mononucleosome fragments. Chromatin was captured using Chromatin Capture Beads and incubated at room temperature for 10 min. End Polishing Master Mix was added and incubated at 22 °C for 30 min, then 65 °C for 30 min. Chromatin ends were ligated using Bridge Ligation Mix and Bridge Ligase, followed by intra-aggregate ligation using Intra-aggregate Ligation Buffer and Enzyme Mix. DNA was isolated by reverse crosslinking with Proteinase K and Crosslink Reversal Buffer, followed by purification. End repair was performed with End Repair Master Mix, followed by adapter ligation using Illumina-compatible adapters, ligation enzyme mix, and ligation enhancer. Libraries were purified with SPRI select beads (Cat # 23319, Beckman Coulter), captured using Streptavidin beads, and amplified by PCR. Size-selected libraries (350–1,000 bp) were sequenced on an Illumina platform.

### 4.2 Chromatin interaction data processing

Micro-C paired-end reads were processed following the Micro-C analysis tutorial provided by Dovetail Genomics with minor adaptations (https://github.com/dovetail-genomics/Micro-C). Sequencing reads were aligned to the mm10 reference genome using BWA-MEM and then processed using *pairtools* (RRID:SCR_023038) (Open2C et al., 2024b) to parse, sort, merge, deduplicate, and classify the ligation products. Library complexity was further assessed using Preseq (RRID:SCR_018664), confirming high diversity of unique read pairs across samples. We applied strict filtering to retain only high-confidence chimeric interactions, ensuring that only uniquely mapped, high-quality interactions were retained. This process removed PCR duplicates and self-ligated artifacts. Chimeric read pairs were defined as those that met any one of three criteria: 1) being located on different chromosomes, 2) having atypical mapping orientations, or 3) being at least 2000 bp apart from each other in linear genomic distance. Valid read pairs were used to generate *.pairs* files for downstream analysis. Chromatin contact matrices were generated from these filtered interaction files using *juicer*_tools (RRID:SCR_017226) to produce .*hic* files and *HiCExplorer* (RRID:SCR_022111) to generate *.cool* files and binned matrix formats at multiple resolutions.

### 4.3 Chromatin interaction analysis

To identify statistically significant chromatin interactions, we analyzed contact matrices at 10 kb resolution using *Fit-Hi-C* (Kaul et al., 2020). The reference genome (mm10) was partitioned into fixed 10 kb bins to generate a genome-wide fragment file. Contact frequencies were extracted from data and processed to create chromosome-specific interaction matrices, including both intra- and inter-chromosomal interactions. These matrices were analyzed to quantify interaction frequencies and compute statistical significance (p-values) for all detected contacts. Significant interactions were identified by applying thresholds based on contact frequency and p-value. For subsequent analyses, we considered interactions passing these thresholds as high-confidence interactions.

### 4.4 Chromatin loop identification

Chromatin loops were identified using the *Mustache* algorithm (RRID:SCR_026110) (https://github.com/ay-lab/mustache) (Roayaei Ardakany et al., 2020) at 5 kb and 10 kb resolutions on *. hic* matrices generated from filtered Micro-C data. These resolutions were selected to capture a range of interaction distances relevant to enhancer-promoter looping and domain-level architecture. Loop distances were categorized into six groups based on genomic span (<200 kb, 200–400 kb, 400–600 kb, 600–800 kb, 800 kb-1 Mb, and >1 Mb) to characterize the distribution of chromatin interactions across scales. Loop anchor positions were used for downstream integration with regulatory element annotations and epigenomic data.

### 4.5 Micro-C data visualization

We performed local and genome-wide analyses using *HiCExplorer* (RRID:SCR_022111) (https://github.com/deeptools/HiCExplorer) (Wolff et al., 2020) to generate interaction matrices and visualize contact intensities across individual chromosomes and selected loci at 20 kb, 10 kb, and 5 kb. Heatmaps were log-transformed and scaled to enable consistent visual comparisons across resolutions and genomic regions. Higher-order features such as A/B compartments, P(s) decay profiles, insulation scores, and TAD boundary structures were analyzed with *cooltools* (RRID:SCR_026118) (https://github.com/open2c/cooltools) (Open2C et al., 2024a) and *HiCexplorer* to characterize genome organization in cochlear cells. For locus-level inspection, chromatin loops detected by *Mustache* were visualized and confirmed using Juicebox (RRID:SCR_021172) (https://github.com/aidenlab/Juicebox), enabling detailed examination of stripes, dots, and loop patterns. To evaluate spatial overlap with disease-associated deletions and epigenomic features, contact maps and loop anchors were integrated with signal tracks in the WashU Epigenome Browser (Li et al., 2019). In addition, we generated heatmaps of ATAC-seq, H3K27ac, H3K4me1, and CTCF signals centered on loop anchors using *deepTools* (RRID:SCR_016366) (https://github.com/deeptools/deepTools), allowing assessment of regulatory mark enrichment at these sites.

### 4.6 ATAC-seq and Cut&Run

ATAC-seq was performed according to previously described protocols with minor modifications (Buenrostro et al., 2013). Briefly, 15,000 viable nuclei were isolated and segmented using the Illumina Tn5 transposase for 30 min at 37 °C. Libraries were amplified with PCR using indexed primers, followed by size selection (100–700 bp) *via* SPRI beads. Paired-end sequencing (2 × 75 bp) was performed on an Illumina NovaSeq platform, yielding ≥50 million reads per sample.

We performed targeted chromatin profiling using CUT&RUN (Skene and Henikoff, 2017) with antibodies against CTCF (Cell Signaling Technology Cat# 3418, RRID:AB_2086791) to map architectural protein binding sites, H3K27ac (Active Motif Cat# 39133, RRID:AB_2561016) to identify active enhancers and promoters, and H3K4me1 (Active Motif Cat# 61781, RRID:AB_3216367) to characterize enhancer regions. For each experiment, 25,000 cells were bound to Concanavalin A-coated beads, permeabilized, and incubated with primary antibodies overnight at 4 °C. Protein A-Micrococcal Nuclease (pA-MNase) fusion protein (1:200 dilution) was subsequently added for 1 h at 4 °C, followed by calcium-activated chromatin digestion (30 min at 0 °C). Purified DNA fragments were processed into sequencing libraries using the Unique Dual Index Kit (Takara Cat #634752) with 12–16 PCR amplification cycles. Paired-end sequencing was performed to achieve >20 million reads per sample. Reads were aligned to the mm10 genome using Bowtie2, and peak calling was performed using stringent thresholds to identify high-confidence binding sites.

### 4.7 Hair cell promoter-enhancer interactions

To examine potential hair cell-specific interactions in our Micro-C data, we utilized *Fit-Hi-C* to collect statistics for the interactions in our whole chimeric dataset and then subset the *Fit-Hi-C* results to keep only interactions taking place with hair cell gene promoters. To achieve this, we utilized a previously published single-cell multiome dataset (GSE182202) (Iyer et al., 2022) from the P1 mouse cochlea to obtain hair cell differentially expressed genes compared to remaining sensory epithelium and surrounding tissues, which consisted of inner phalangeal/border cells, Deiters’ and pillar cells, assorted sulcus cells and interdental cells, Hensen’s and Claudius cells, medial and lateral Kölliker’s organ, and other miscellaneous cells types. *Seurat* (https://github.com/satijalab/seurat) (RRID:SCR_007322) was used to calculate positive DEGs with a log fold-change threshold of 3 and a minimum expression of 25% in hair cells, giving us 270 genes. From a BED file containing transcription start sites with gene symbol annotations from the mm10 genome, we prepared a new BED file subset by the list of 270 hair cell genes. We then turned this hair cell TSS BED file into a new BED file covering gene promoter regions. To define the coordinates of the promoter regions, we overlapped a macs2 peaks BED file from our bulk ATAC purified mouse P1 hair cell dataset with the transcription start sites. Any promoters whose TSS did not overlap with any peaks were assigned coordinates spanning 2000 bp upstream of the TSS and 500 bp downstream of the TSS. We utilized this new promoter BED file to subset our *Fit-Hi-C* results to keep interactions where at least one of the regions overlapped with a hair cell gene promoter. *Fit-Hi-C* results consist of a fragment midpoint (single base pair locus) within a bin. Thus, to define the regions to overlap with the promoter file, we extended the midpoint by half the bin size both upstream and downstream. We also annotated each interacting region with the gene symbol of the promoter(s) with which it overlapped. *Fit-Hi-C* was run at resolutions of 10, 5, 2 and 1 kb and all results were examined thoroughly. We used this as a general guide to point us to some examples of the most significant and frequently occurring interactions, though plenty of genes we examined that are enriched in hair cells did not meet the DEGs criteria and were not included in the list of 270. For our Supplementary Table S5, containing subset HC promoter-enhancer interactions, we also extracted interactions between the known *Atoh1* downstream enhancers from the original unfiltered *Fit-Hi-C* results, even if some of the interactions between these enhancers did not overlap with the *Atoh1* promoter.

## Data Availability

The data presented in the study are deposited in the NCBI GEO, accession number GSE305205.
