# Supplementary figures and images for: Mapping of chromatin architecture and enhancer-promoter interactions in the cochlea
Source: Front Mol Biosci. 2025 Oct 15;12:1683964. doi: 10.3389/fmolb.2025.1683964 (PMC12568336; doi:10.3389/fmolb.2025.1683964)

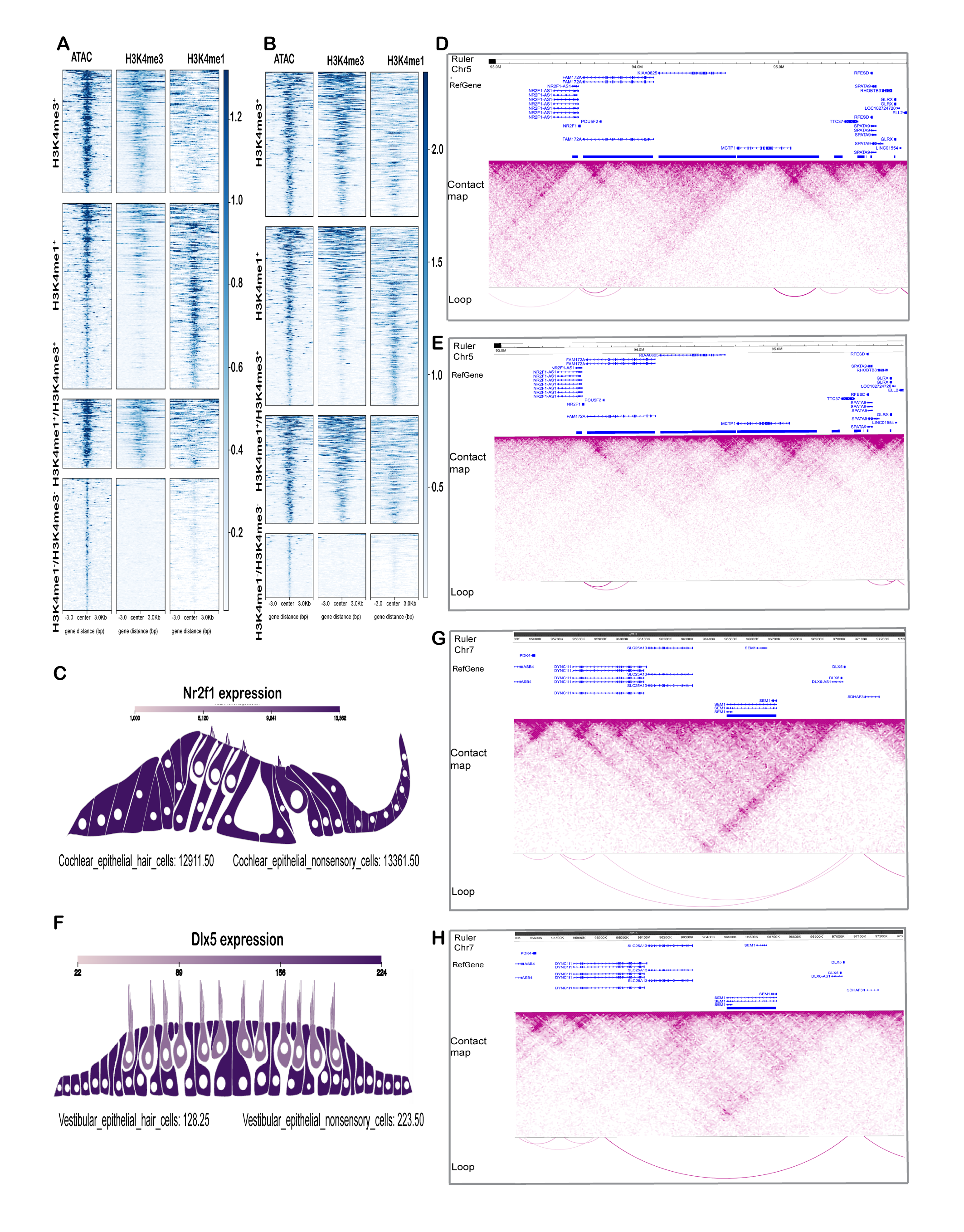

Supplement: Supplementary file 1 [file Image6.tif]

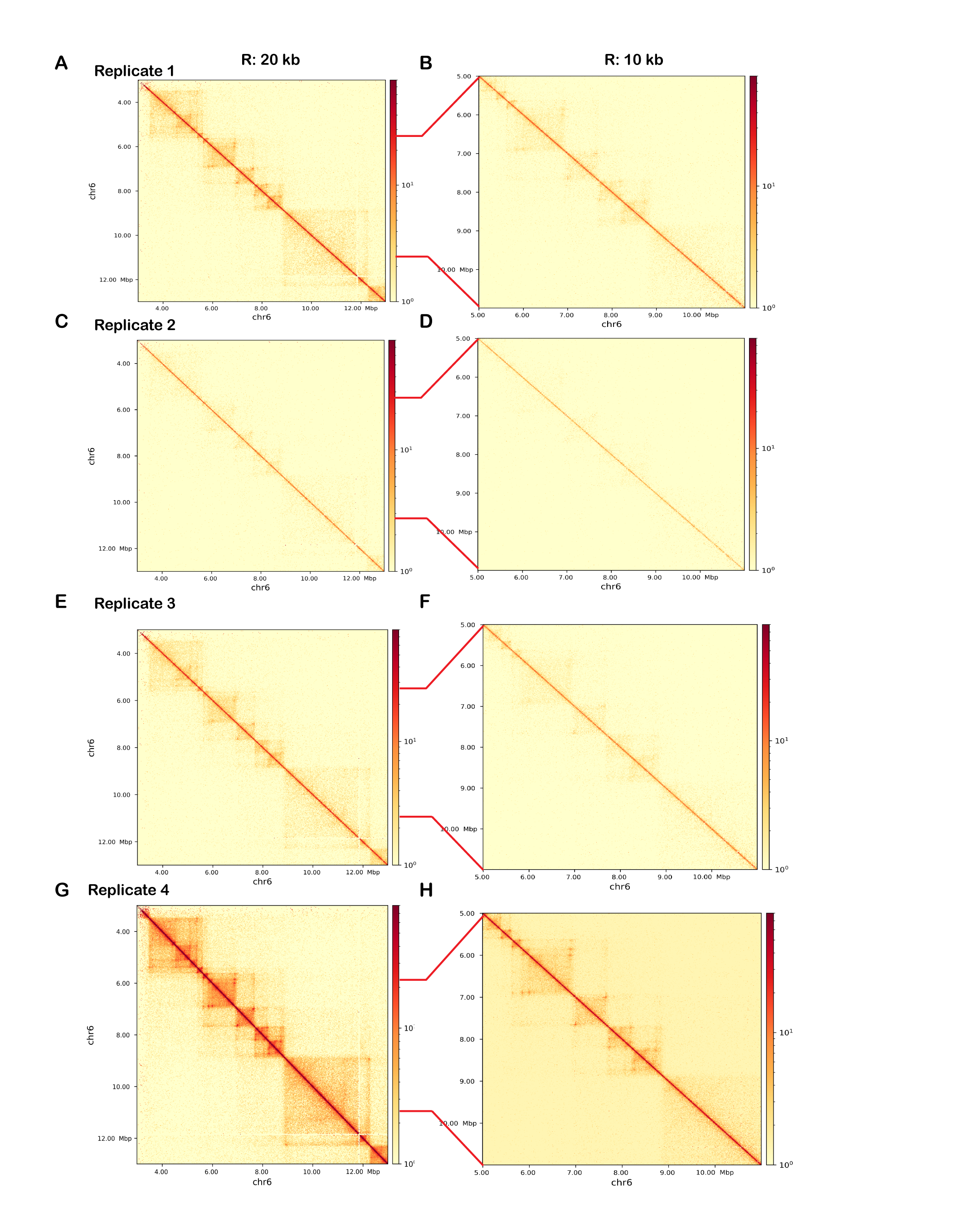

Supplement: Supplementary file 3 [file Image3.tif]

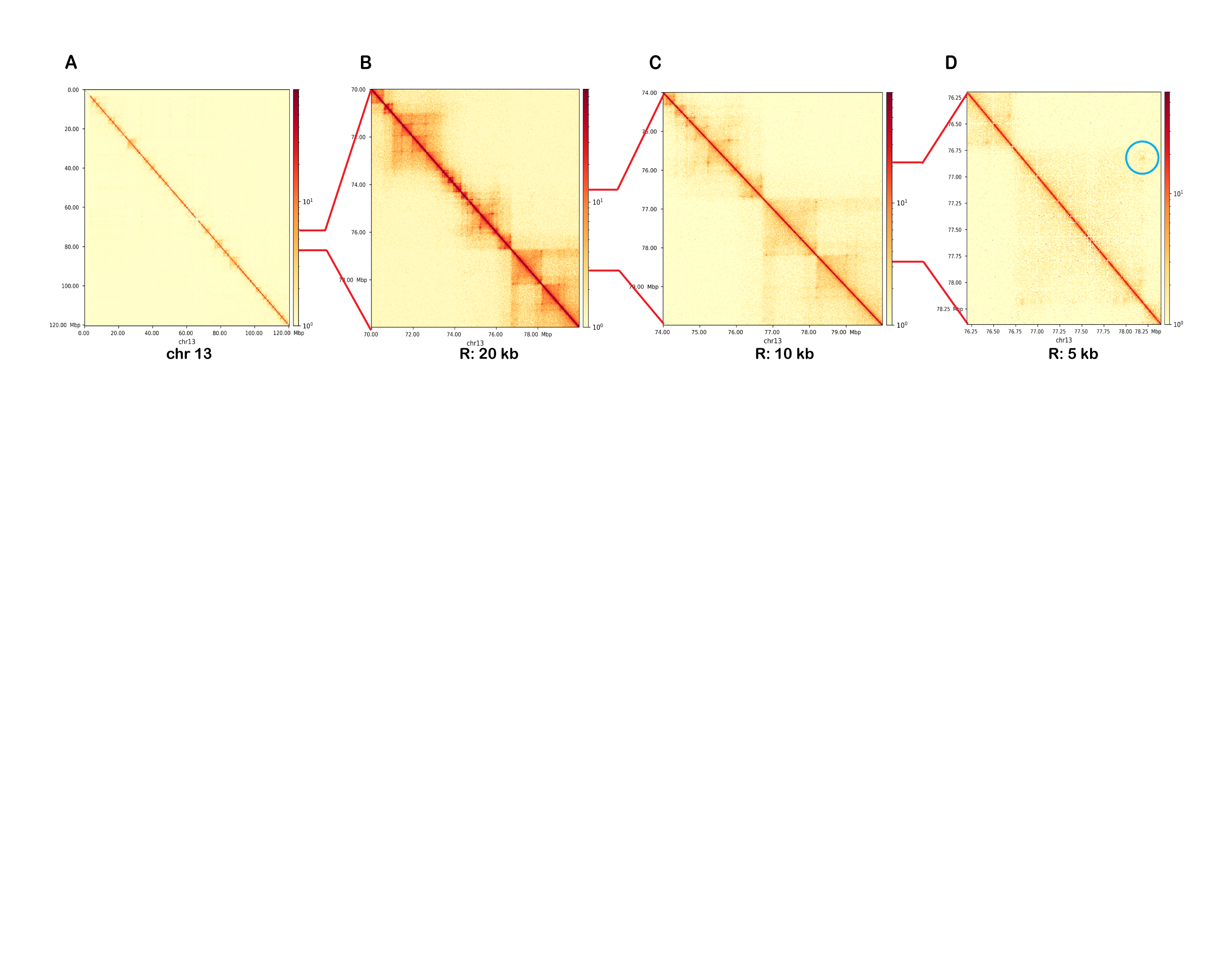

Supplement: Supplementary file 5 [file Image4.tif]

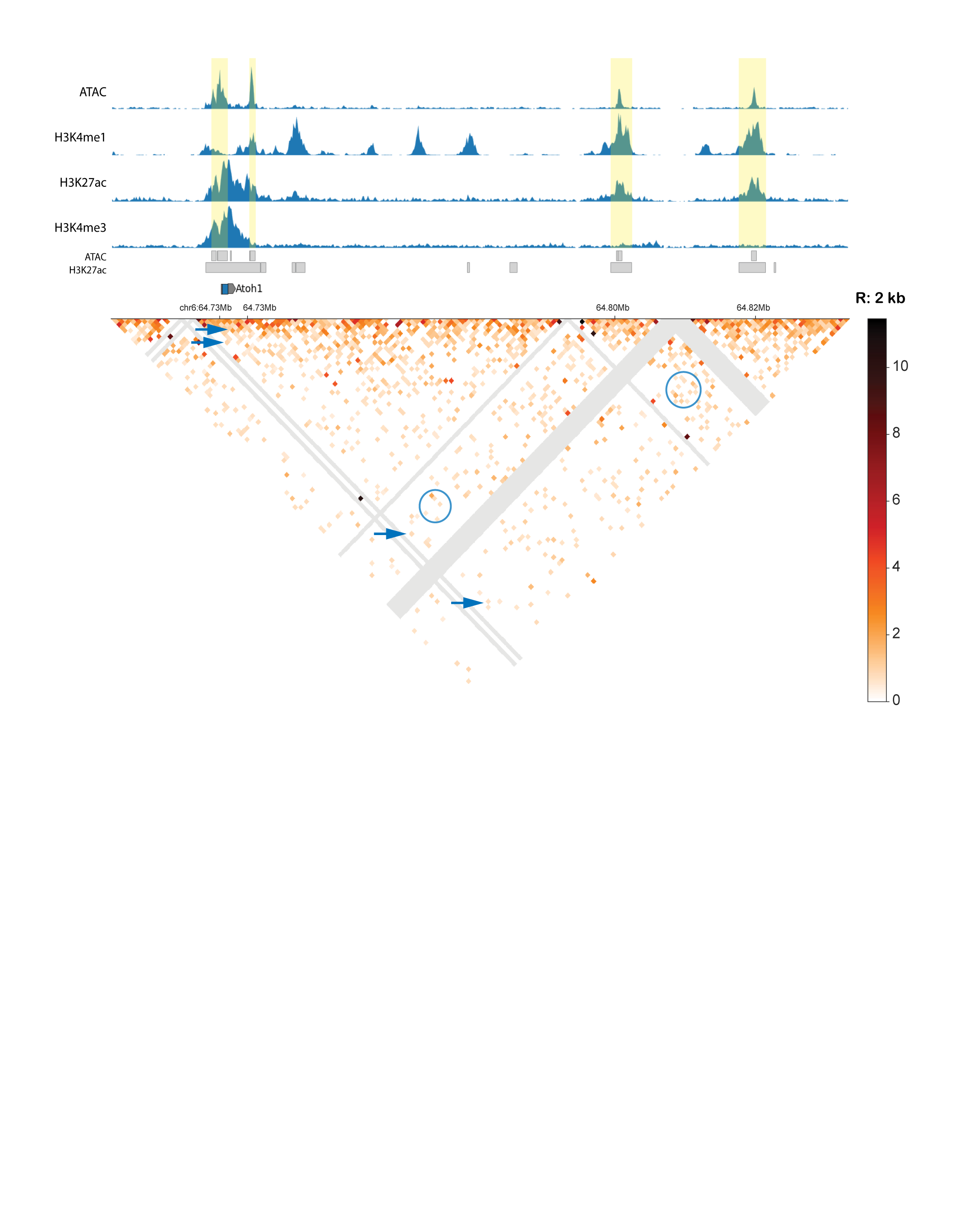

Supplement: Supplementary file 6 [file Image9.tif]

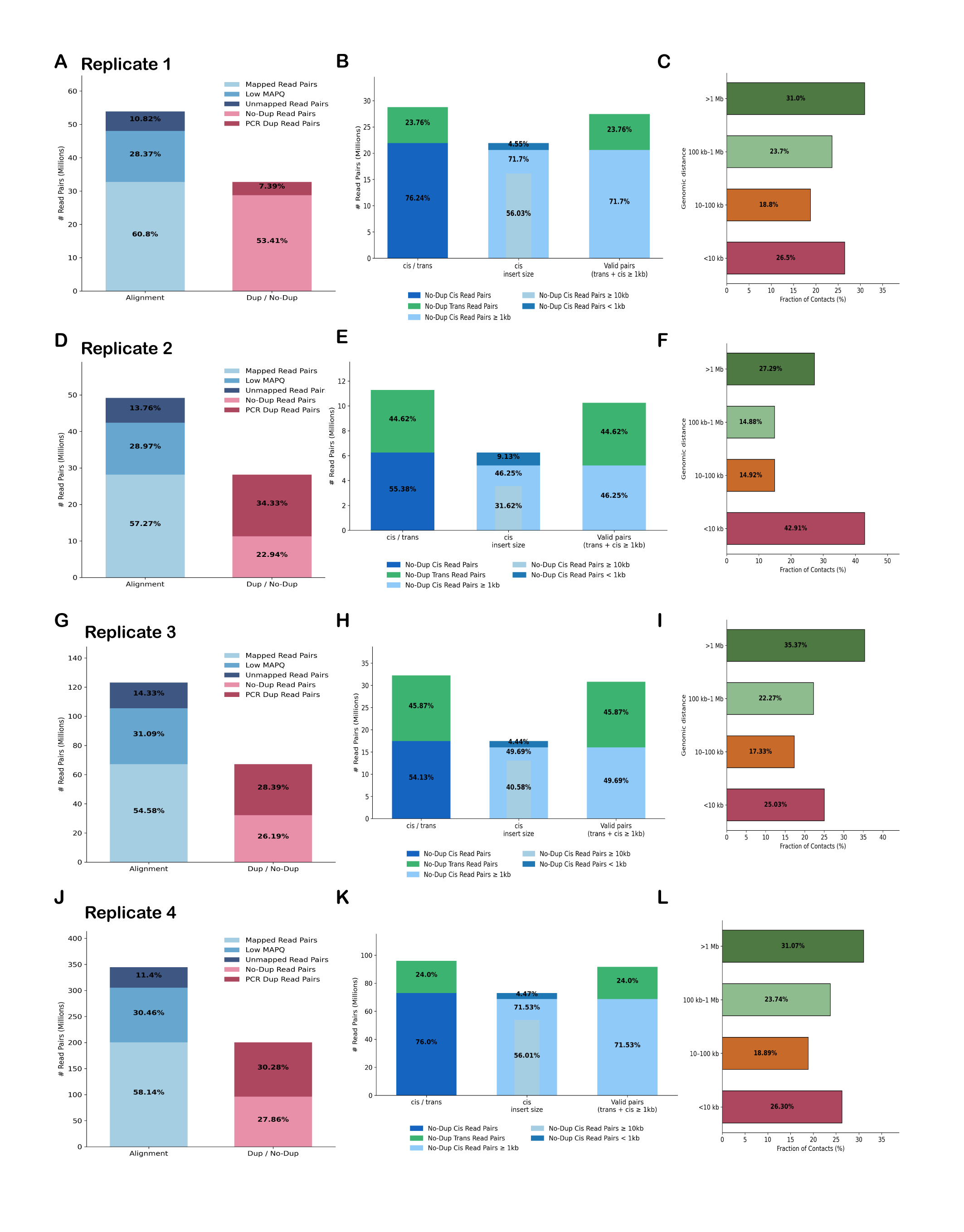

Supplement: Supplementary file 7 [file Image2.tif]

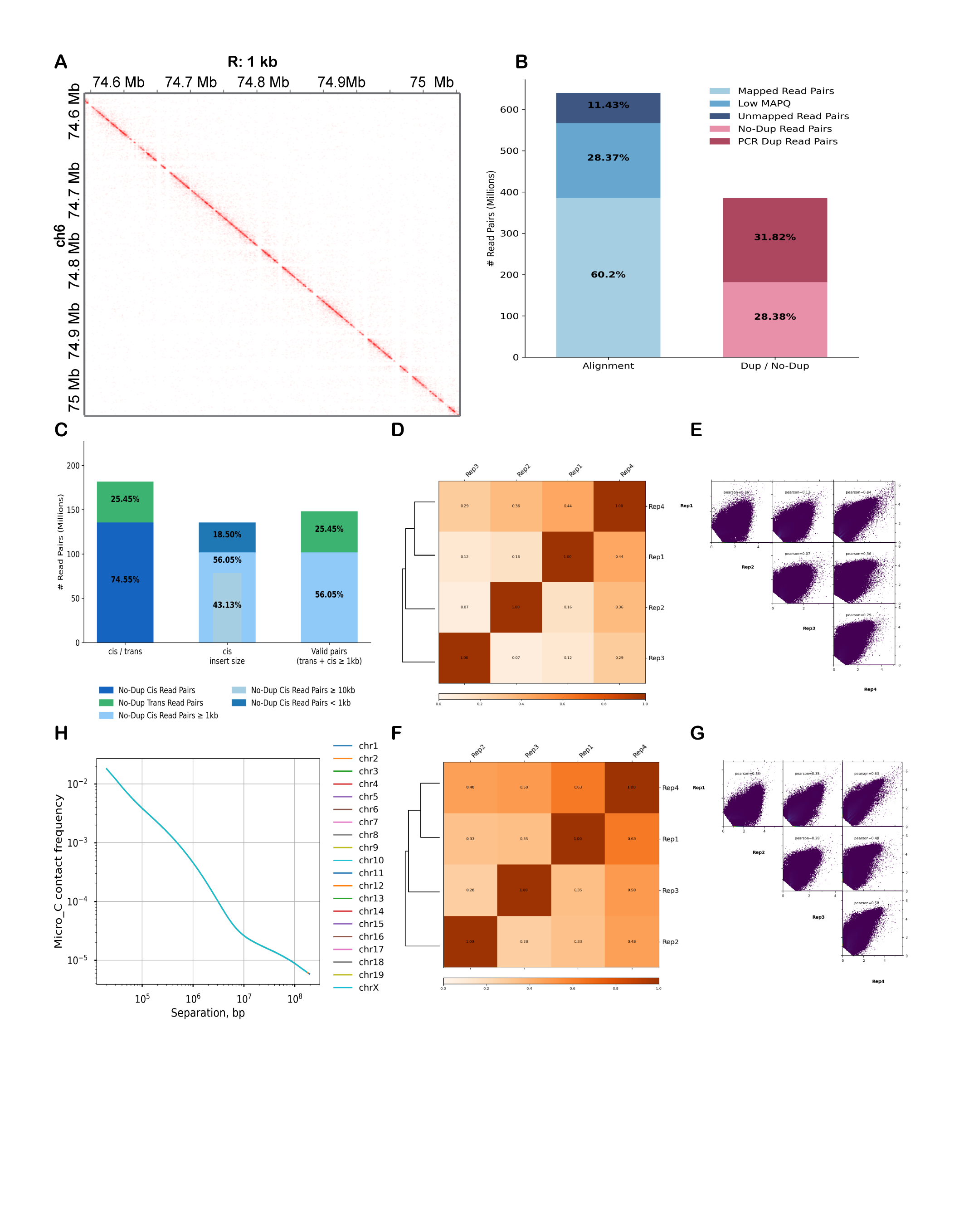

Supplement: Supplementary file 8 [file Image1.tif]

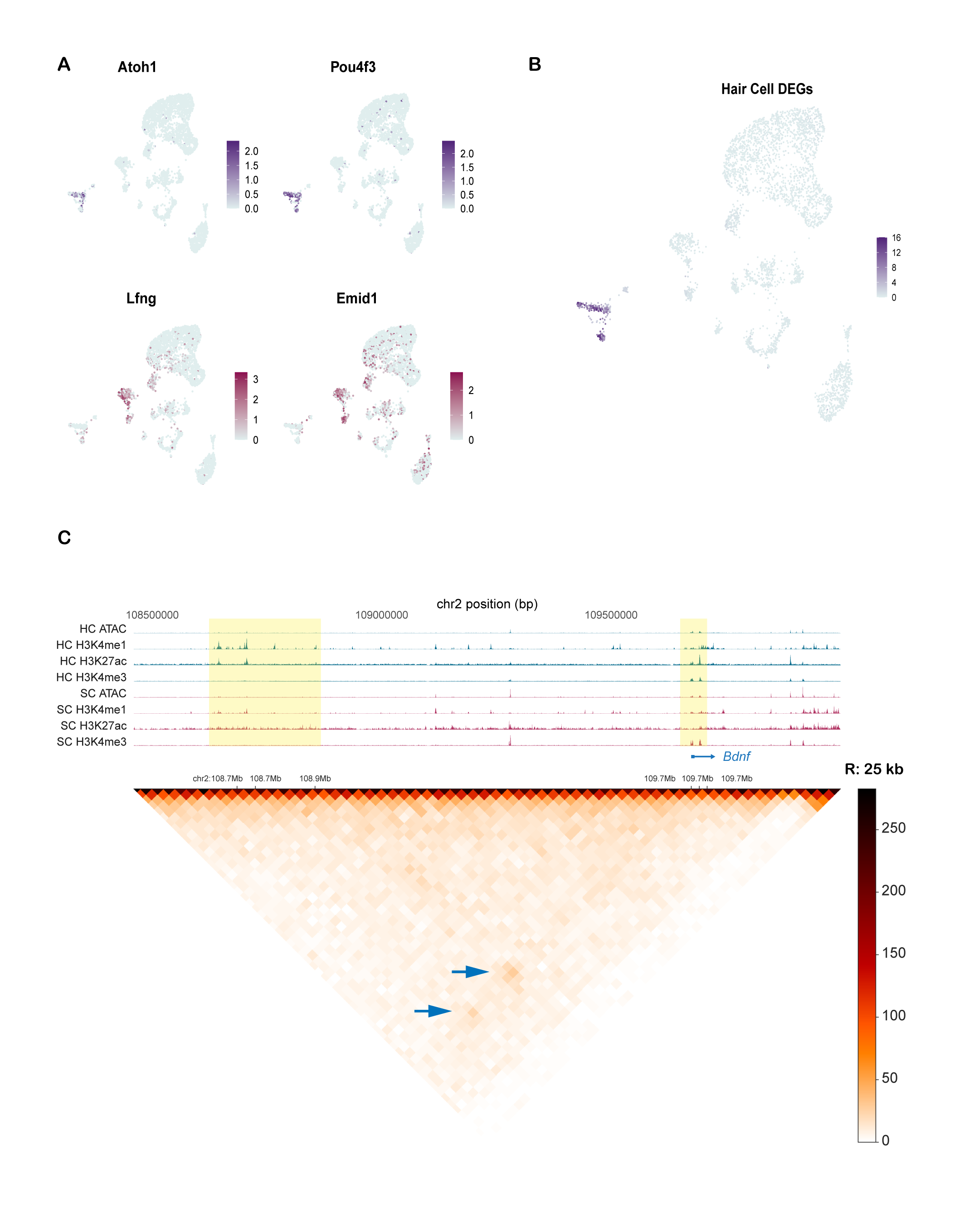

Supplement: Supplementary file 9 [file Image7.tif]

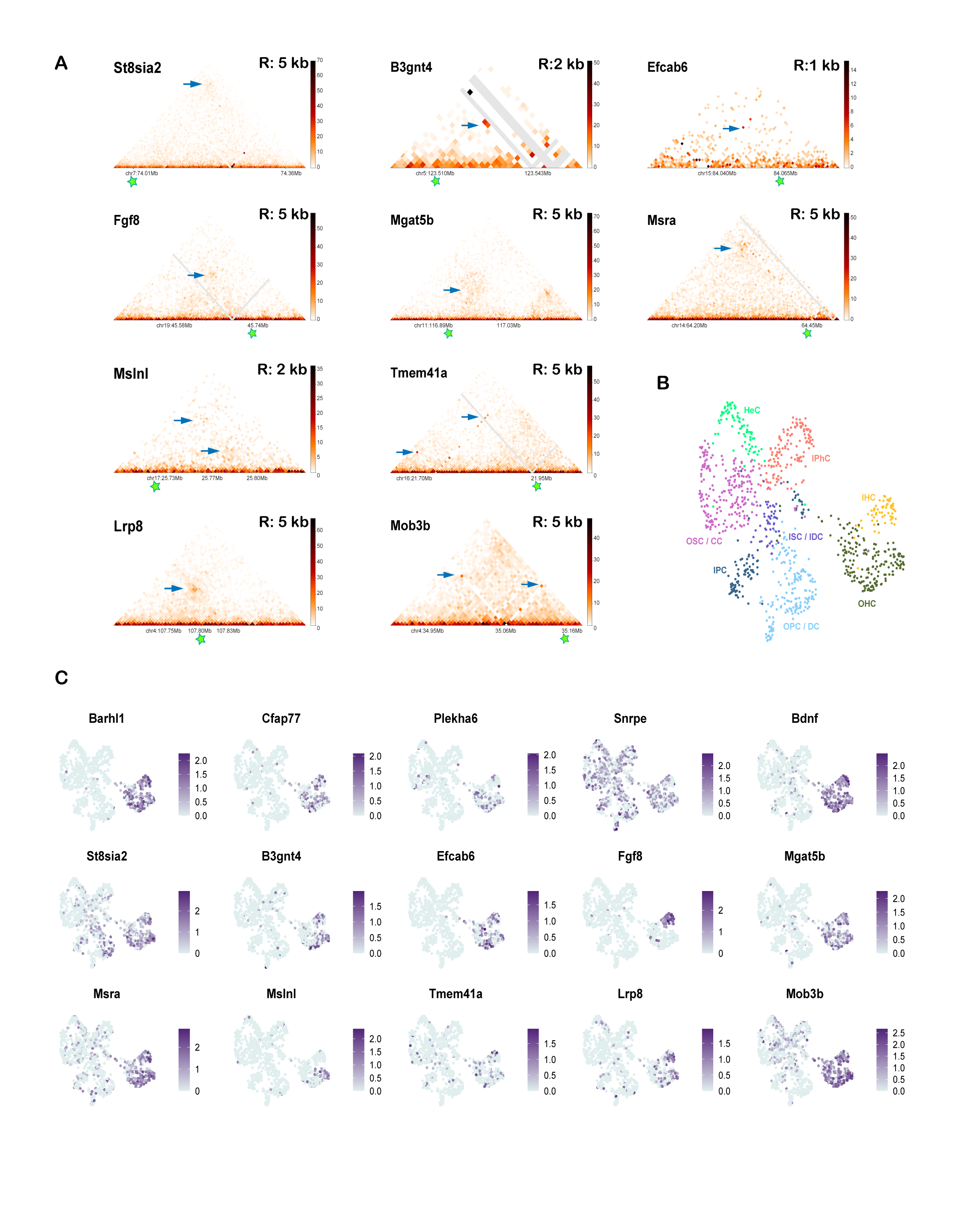

Supplement: Supplementary file 11 [file Image8.tif]

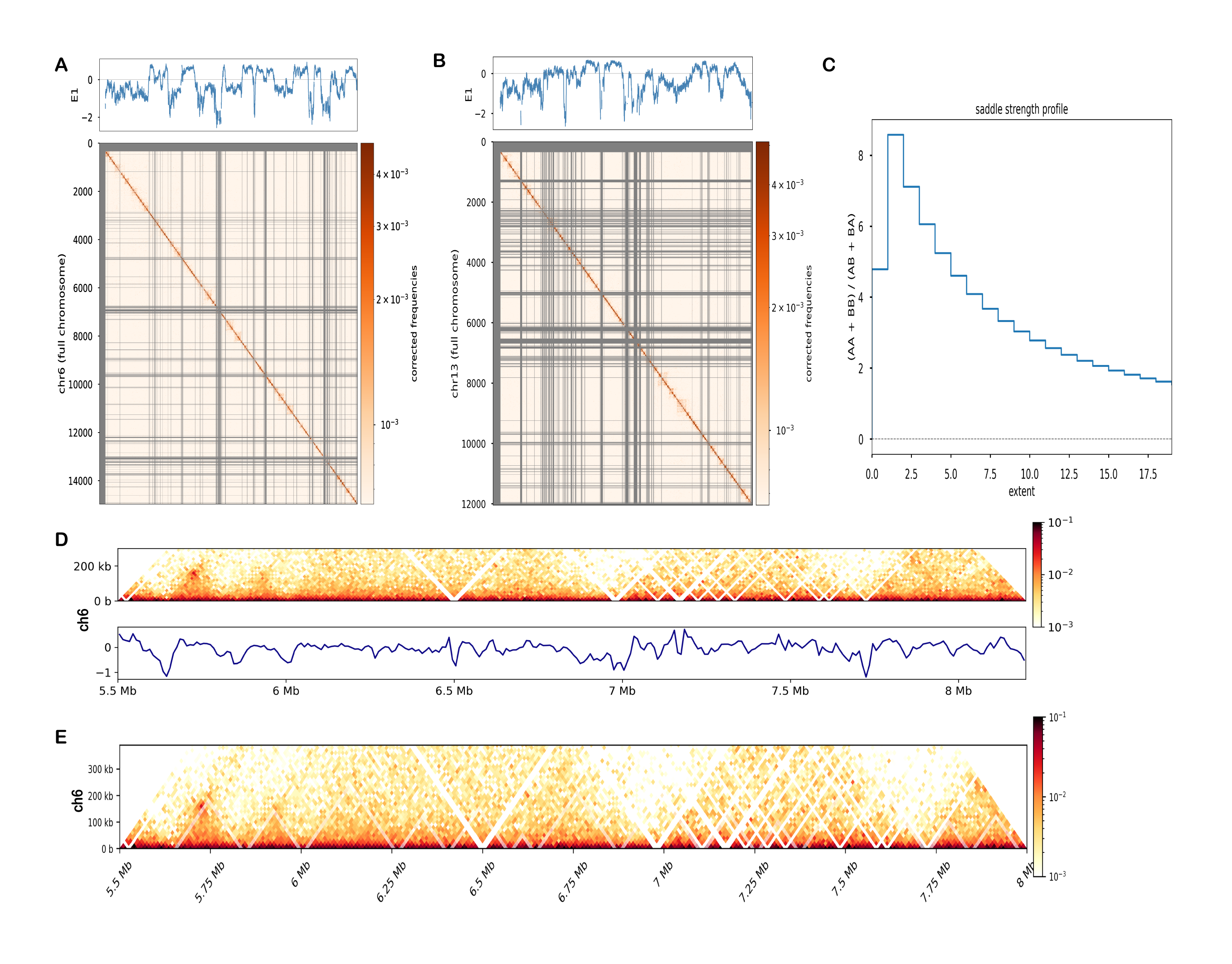

Supplement: Supplementary file 12 [file Image5.tif]
