# Supplementary material for: Mapping of chromatin architecture and enhancer-promoter interactions in the cochlea
Source: Front Mol Biosci. 2025 Oct 15;12:1683964. doi: 10.3389/fmolb.2025.1683964 (PMC12568336; doi:10.3389/fmolb.2025.1683964)
Supplement: Supplementary file 2 [file Supplementaryfile1.docx]

Supplementary Material

# Supplementary Data

# Supplementary Figures and Tables

## Supplementary Figures

**Supplementary Figure 1.** **Unfiltered contact maps and extended quality metrics.** **(A)** Raw, unfiltered contact map for a representative region of chromosome 6, 1 kb resolution (74.6-75.1 Mb). Dense signals from self-ligation and short-range artifacts are visible as background noise. **(B)** Read processing summary for the unfiltered dataset. Categories include mapped reads, low mapping quality (MAPQ), unmapped reads, PCR duplicates, and non-duplicate read pairs. **(C)** Interaction classification for unfiltered data. Bar plots display proportions of cis versus trans contacts, as well as the fraction of cis reads spanning ≥1 kb compared to short-range (<1 kb) contacts. **(D-G)** Pearson correlation heatmaps (D, 10 kb; F, 20 kb) and pairwise scatter plots (E, 10 kb; G, 20 kb) of Hi-C contact matrices across biological replicates. **(H)** After smoothing and normalization, the decay of contact probability P(s) with genomic distance across all chromosomes.

**Supplementary Figure 2. Summary of sequencing, alignment, and interaction classification metrics across all four biological replicates.** **(A, D, G, J)** Stacked bar plots showing read pair processing statistics for replicates 1-4, respectively. **(B, E, H, K)** Classification of non-duplicate read pairs from replicates 1-4. **(C, F, I, L)** Distribution of genomic distances spanned by cis interactions for replicates 1-4, grouped into bins: <10 kb, 10-100 kb, 100 kb-1 Mb, and >1 Mb.

**Supplementary Figure 3**. **Multi-resolution contact maps on chromosome 6 across biological replicates.** (A-H) Normalized contact matrices for Replicates 1-4 at 20 kb resolution (A, C, E, G) and 10 kb resolution (B, D, F, H). Each panel shows intra-chromosomal interaction frequencies on a log scale. Red boxes indicate the zoomed-in region shown at higher resolution in the adjacent panels.

**Supplementary Figure 4. Multi-scale intra-chromosomal architecture of chromosome 13.** **(A)** Chromosome-wide interaction map for chromosome 13, showing the full range of cis contacts along the chromosome. **(B)** Zoomed-in region of chromosome 13 (72-80 Mb) at 20 kb resolution. **(C)** Further magnification within chromosome 13 (74-80 Mb) at 10 kb resolution. **(D)** High resolution view of chromosome 13 (74.5-78.3 Mb) at 5 kb resolution.

**Supplementary Figure 5. Extended analysis of chromatin compartments and TAD boundaries.** **(A)** Normalized Micro-C contact matrix for chromosome 6 and **(B)** chromosome 13 with the corresponding E1 profile, showing alternating A/B compartments across the chromosome. **(C)** Saddle strength profile quantifying compartmentalization as a function of genomic distance. Same compartment interactions (A-A and B-B) dominate at short distances and gradually decay with increasing extent. **(D)** Example region on chromosome 6 (5.5-8.5 Mb) with 10 kb binned contact matrix overlaid with insulation scores (blue line). **(E)** Higher resolution view of the same region, highlighting consistent TAD structures and boundaries, confirming the segmentation of cochlear chromatin into individual domains with white lines.

**Supplementary Figure 6. Cell type-specific chromatin states at loop anchors and validation of locus-level loops. (A)** Heatmaps of ATAC-seq, H3K4me3, and H3K4me1 signals centered on HC and **(B)** SC, revealing distinct enhancer-like (H3K4me1⁺), promoter-like (H3K4me3⁺), and mixed chromatin states, compared to anchors lacking both marks. **(C)** Gene expression plot from gEAR showing *Nr2f1* expression across cochlear epithelial hair cells and non‑sensory cells. **(D)** WashU Epigenome Browser view of the *MCTP1*-*NR2F1* locus (10 kb) using non-cochlear human motor neurons and **(E)** GM12878 lymphoblastoid cells. **(F)** Gene expression plot from gEAR showing *Dlx5* expression. **(G)** WashU Epigenome Browser view of the *DYNC1I1*-*DLX5/6* locus (10 kb) in human motor neurons and **(H)** GM12878 lymphoblastoid cells.

**Supplementary Figure 7. Expression of genes in scMultiome dataset that represent significant interactions in Micro-C dataset. (A)** Gene markers indicate the identities of hair cells and supporting cell groups in single-cell multiome dataset. **(B)** Aggregated expression of hair cell differentially expressed genes in single-cell multiome dataset after QC. **(C)** Interactions of distal enhancers with *Bdnf* promoter region, binned at 25kb. Blue arrows indicate a stronger signal representing the intersection of interacting loci. Translucent yellow regions in coverage tracks depict approximate spans of interacting regions. Tick marks on the heatmap represent some midpoints determined by *Fit-Hi-C*.

**Supplementary Figure 8. Hair cell promoter-DRE interactions in the Micro-C dataset.** **(A)** Heatmaps of some of the top differentially expressed genes in hair cells compared to the remaining SE cell types. (Resolutions used: *St8sia2* - 5 kb; *B3gnt4* - 2 kb; *Efcab6* - 1 kb; *Fgf8* - 5 kb; *Mgat5b* - 5 kb; *Msra* - 5 kb; *Mslnl* - 2 kb; *Tmem41a* - 5 kb; *Lrp8* - 5 kb; *Mob3b* - 5 kb.) Blue arrows indicate the intersection of two genomic loci that are interacting in 3D space. Grey space indicates that no data exists for this region. Scale bars measure contact counts. Green stars indicate the transcription start site of the gene of interest. **(B)** Cluster identities in single-cell multiome dataset, subset to include hair cells, supporting cells, and a few other cell types for easy viewing. **(C)** Expression of hair cell DEGs whose promoters appeared in interactions in Micro-C dataset. UMAP plots represent single-cell multiome dataset subset to include hair cells, supporting cells, and a few other cell types for easy viewing. OHC = Outer Hair Cell; IHC = Inner Hair Cell; IPhC = Inner Phalangeal Cell; IPC = Inner Pillar Cell; OPC / DC = Outer Pillar Cell / Deiters’ Cell; ISC / IDC = Inner Sulcus Cell / Interdental Cell; OSC / CC = Outer Sulcus Cell / Claudius Cell; HeC = Hensen’s Cell.

**Supplementary Figure 9. Interactions between known *Atoh1* downstream enhancers and promoter regions in Micro-C data.** Tracks include bigwig signal files for ATAC and various histone modification CUT&RUN (top four), and BED files containing peaks identified by macs2 for both ATAC and H3K27 acetylation CUT&RUN (bottom two). All tracks represent purified mouse hair cells. Yellow highlighted bars identify the promoter region and enhancers #1-3 (left to right). Signal strength in the heatmap (2 kb) is depicted by contact frequency (darker signals = more interactions between loci). The blue circles and arrows identify the intersection of two interacting loci.

## Supplementary Tables

## Supplementary Table 1: Summary of Micro-C read pairs and interaction classifications prior to filtering (unfiltered dataset).

| **Category** | **Count** | **Percent** |
| --- | --- | --- |
| Total Read Pairs | 640,114,904 | 100.00% |
| Unmapped Read Pairs | 73,148,252 | 11.43% |
| Mapped Read Pairs | 385,370,373 | 60.20% |
| PCR Dup Read Pairs | 203,691,492 | 31.82% |
| No-Dup Read Pairs | 181,678,881 | 28.38% |
| No-Dup Cis Read Pairs | 135,438,455 | 74.55% |
| No-Dup Trans Read Pairs | 46,240,426 | 25.45% |
| No-Dup Valid Read Pairs (cis >= 1kb + trans) | 148,065,509 | 81.50% |
| No-Dup Cis Read Pairs < 1kb | 33,613,372 | 18.50% |
| No-Dup Cis Read Pairs >= 1kb | 101,825,083 | 56.05% |
| No-Dup Cis Read Pairs >= 10kb | 78,361,020 | 43.13% |

Proportion of Total Read Pairs

Proportion of No-Dup Read Pairs

## Supplementary Table 2: Summary of sequencing and mapping statistics for four biological replicates. Each metric is presented as the absolute count followed by the percentage relative to the total read pairs for the corresponding replicate.

| **Metric** | **Replicate 1** | **Replicate 2** | **Replicate 3** | **Replicate 4** |
| --- | --- | --- | --- | --- |
| **Total Read Pairs** | 53,862,367 (100%) | 49,175,007 (100%) | 123,112,580 (100%) | 344,568,051 (100%) |
| **Unmapped Read Pairs** | 5,828,060 (10.82%) | 6,768,438 (13.76%) | 17,637,389 (14.33%) | 39,292,339 (11.4%) |
| **Mapped Read Pairs** | 32,748,180 (60.8%) | 28,161,760 (57.27%) | 67,198,585 (54.58%) | 200,319,908 (58.14%) |
| **PCR Dup Read Pairs** | 3,978,922 (7.39%) | 16,881,140 (34.33%) | 34,952,775 (28.39%) | 104,324,851 (30.28%) |
| **No-Dup Read Pairs** | 28,769,258 (53.41%) | 11,280,620 (22.94%) | 32,245,810 (26.19%) | 95,995,057 (27.86%) |
| **No-Dup Cis Read Pairs** | 21,934,407 (76.24%) | 6,247,234 (55.38%) | 17,453,629 (54.13%) | 72,952,625 (76.0%) |
| **No-Dup Trans Read Pairs** | 6,834,851 (23.76%) | 5,033,386 (44.62%) | 14,792,181 (45.87%) | 23,042,432 (24.0%) |
| **No-Dup Valid Read Pairs (cis ≥1kb + trans)** | 27,461,503 (95.45%) | 10,250,865 (90.87%) | 30,814,749 (95.56%) | 91,708,512 (95.53%) |
| **No-Dup Cis Read Pairs < 1kb** | 1,307,755 (4.55%) | 1,029,755 (9.13%) | 1,431,061 (4.44%) | 4,286,545 (4.47%) |
| **No-Dup Cis Read Pairs ≥ 1kb** | 20,626,652 (71.7%) | 5,217,479 (46.25%) | 16,022,568 (49.69%) | 68,666,080 (71.53%) |
| **No-Dup Cis Read Pairs ≥ 10kb** | 16,118,022 (56.03%) | 3,566,647 (31.62%) | 13,085,196 (40.58%) | 53,768,761 (56.01%) |

**Supplementary Table 3: Supplementary Table 3. Chromatin interactions identified by Mustache, with observed/expected (O/E) ratios.** The table reports the interacting loci (chromosome and genomic coordinates), statistical significance (FDR), detection scale, and observed/expected interaction ratio (O/E, from *Fit-Hi-C* at 10 kb resolution). NA indicates cases where *Fit-Hi-C* did not report a significant interaction at the corresponding bin pair. The table includes all interactions passing with FDR < 0.1.

## Supplementary Table 4: Genomic distance distribution of significant chromatin loops. Number of loops identified across genomic distance bins, filtered at FDR < 0.1 and FDR < 0.05 thresholds.

| **Distance Bin** | **FDR < 0.1** | **FDR < 0.05** |
| --- | --- | --- |
| <200 kb | 2,324 | 1,846 |
| 200–400 kb | 2,141 | 1,671 |
| 400–600 kb | 1,035 | 825 |
| 600–800 kb | 534 | 408 |
| 800 kb – 1 Mb | 328 | 266 |
| >1 Mb | 436 | 340 |
| **Total Loops** | **6,798** | **5,356** |

## Supplementary Table 5: Multiple resolution *Fit-Hi-C* results subset to a list of hair cell gene promoters and *Atoh1* 3’ enhancers.

**Supplementary Table 6: GREAT Gene Ontology analysis of the top 3000 interacting regions for either inter- or intra-chromosomal interactions.**
